# Sex-Dependent Effects of Intestinal Microbiome Manipulation in a Mouse Model of Alzheimer’s Disease

**DOI:** 10.3390/cells10092370

**Published:** 2021-09-09

**Authors:** Harpreet Kaur, Suba Nookala, Surjeet Singh, Santhosh Mukundan, Kumi Nagamoto-Combs, Colin Kelly Combs

**Affiliations:** 1 Department of Biomedical Sciences, University of North Dakota, School of Medicine and Health Sciences, 1301 N Columbia Road, Grand Forks, ND 58202-9037, USA; suba.nookala@und.edu (S.N.); santhosh.mukundan@und.edu (S.M.); kumi.combs@und.edu (K.N.-C.); 2 Department of Neuroscience, Canadian Centre for Behavioural Neuroscience (CCBN), University of Lethbridge, 4401 University Drive, Lethbridge, AB T1K 3M4, Canada; surjeet.singh@uleth.ca

**Keywords:** Alzheimer’s disease, probiotic, VSL#3, antibiotics, synbiotic, prebiotic, microbiome, cytokines, splenocytes, immune response

## Abstract

Mechanisms linking intestinal bacteria and neurodegenerative diseases such as Alzheimer’s disease (AD) are still unclear. We hypothesized that intestinal dysbiosis might potentiate AD, and manipulating the microbiome to promote intestinal eubiosis and immune homeostasis may improve AD-related brain changes. This study assessed sex differences in the effects of oral probiotic, antibiotics, and synbiotic treatments in the *App^NL-G-F^* mouse model of AD. The fecal microbiome demonstrated significant correlations between bacterial genera in *App^NL-G-F^* mice and Aβ plaque load, gliosis, and memory performance. Female and not male *App^NL-G-F^* mice fed probiotic but not synbiotic exhibited a decrease in Aβ plaques, microgliosis, brain TNF-α, and memory improvement compared to no treatment controls. Although antibiotics treatment did not produce these multiple changes in brain cytokines, memory, or gliosis, it did decrease Aβ plaque load and colon cytokines in *App^NL-G-F^* males. The intestinal cytokine milieu and splenocyte phenotype of female but not male *App^NL-G-F^* mice indicated a modest proinflammatory innate response following probiotic treatment compared to controls, with an adaptive response following antibiotics treatment in male *App^NL-G-F^* mice. Overall, these results demonstrate the beneficial effects of probiotic only in *App^NL-G-F^* females, with minimal benefits of antibiotics or synbiotic feeding in male or female mice.

## Highlights

1. Probiotic feeding reduced Aβ plaque load and improved memory in *App^NL-G-F^* female mice.
2. Probiotics decreased microgliosis and TNF-α levels in *App^NL-G-F^* female mice with no effects in male mice.
3. Oral antibiotic treatment reduced Aβ plaque load in *App^NL-G-F^* male mice with no effects in female mice.
4. Probiotic feeding stimulated innate immune response changes in *App^NL-G-F^* female spleens.
5. Antibiotic treatment stimulated acquired immune response changes in *App^NL-G-F^* male spleens.

## 1. Introduction

Accumulating evidence suggests that bidirectional communication between the central nervous system (CNS) and the enteric nervous system (ENS) and the gut microbiota plays a central role in this interaction. The gut microbiota performs functions including metabolizing food and drugs [1,2], providing nutrients from dietary carbohydrates [3,4], maintaining the gut barrier integrity [5], immunomodulation [6], and protecting against pathogens [7], thus helping to maintain a stable gut ecosystem [8]. Alterations in the composition of gut microbiota, or dysbiosis, is associated with various gastrointestinal disorders such as inflammatory bowel disease and colon cancer [9,10], as well as extra-intestinal diseases such as metabolic syndrome, obesity, allergies, autism, anxiety, depression, and neurodegenerative diseases [11,12,13]. In addition, recent advances in research of Alzheimer’s disease (AD) etiology points towards an altered gut microbiota leading to a peripheral/systemic inflammatory response that affects brain functions [14,15,16]. Although the mechanisms are unclear, numerous complex interactions between intestinal microbiota and the immune system likely influence intestinal, circulating, and brain immune cells in a fashion contributing to AD [17,18]. 

The intestinal microbiota has a profound impact on shaping the host immune system. It regulates both the innate and adaptive immune systems by stimulating B cell and antibody responses and the differentiation of T helper cells [19]. Recently, an association of specific bacterial species with the development of particular T cell subtypes has been found. For example, segmented filamentous bacteria in the gut can promote inflammatory bowel diseases, experimental autoimmune encephalomyelitis, and autoimmune arthritis by inducing Th17 cell differentiation [20,21]. On the other hand, the spore-forming component of indigenous intestinal microbiota belonging to the genus *Clostridium* promotes the accumulation of regulatory T cells (Tregs), which are anti-inflammatory and prevent gut inflammation [22]. Neuroimmune modulation by the microbiota regulates responses to neuroinflammation, brain injury, autoimmunity, and neurogenesis and, therefore, contributes to the etiopathogenesis or manifestation of neurodevelopmental, psychiatric, and neurodegenerative diseases [23].

Several experimental approaches have been used in animal models to understand the effects of gut microbiota in diseases, including germ-free mouse models, manipulation of gut microbiota with antibiotics, fecal microbial transplantation, and administration of probiotics or prebiotics [24]. We have previously published that the gut microbiota is involved in AD pathophysiology, and probiotic intervention reduces intestinal permeability, inflammation, and anxiety in the *App^NL-G-F^* mouse AD model [25,26]. Probiotics are live microorganisms that confer a health benefit on the host when administered at adequate amounts. They are known for alleviating gastrointestinal symptoms, enhancing gut-barrier functions, protecting against infectious diseases by blocking pathogenic bacteria, competing with pathogens and toxins for adherence to the intestinal epithelium, enhancing gut-barrier functions, and, most importantly, modulation of the immune system [27,28].

In this study, we provided a combination of two broad-spectrum non-absorbable antibiotics, vancomycin and neomycin, along with an anti-fungal, pimaricin, (ABX) in the drinking water for ten days to C57BL/6J and *App^NL-G-F^* mice to deplete intestinal bacterial communities, followed by treatment with either a probiotic or a prebiotic in their diet for 60 days. Two other groups of animals were treated only with antibiotics for ten days or only with VSL#3 for 60 days. Overall, we observed that probiotic and antibiotic interventions significantly altered fecal microbiota relative to controls, and response to interventions was significantly different between sexes. In particular, we observed that *App^NL-G-F^* females with VSL#3 supplementation had significantly fewer Aβ plaques, lower gliosis, and lower brain cytokine levels and improved memory relative to control animals. No effect of VSL#3 supplementation was observed in male *App^NL-G-F^* animals, although they showed reduced Aβ plaques following antibiotics treatment. These changes correlated with a probiotic-dependent increase in colonic Th2 associated cytokines and activated splenic macrophages in *App^NL-G-F^* females. An opposite trend was observed in *App^NL-G-F^* males, where probiotic treatment had minimal effects, but antibiotics treatment increased colonic levels of Th1, Th2, and Th17 cytokines and increased splenic T cells. Thus, our study demonstrates a differential innate versus adaptive immunomodulatory impact of probiotic and broad-spectrum antibiotics on female versus male *App^NL-G-F^* mice, respectively.

## 2. Materials and Methods

### 2.1. Animals

*App^NL-G-F^* mice were obtained from Dr. Takaomi C. Saido, Laboratory for Proteolytic Neuroscience, RIKEN Center for Brain Science, Japan. APP is not overexpressed in *App^NL-G-F^* mice, but pathogenic Aβ is elevated due to effects from three mutations associated with familial AD. An APP construct containing a humanized Aβ region, which includes the Swedish “NL”, the Iberian “F”, and the Arctic “G” mutations, was used [29]. This model was selected to avoid potential artifacts introduced by APP overexpression. A total of 70 male and female *App^NL-G-F^* (AD) and 70 male and female C57BL/6J (wild type, WT) mice (total 140 mice) were used at 2–3 months of age. Five to seven mice were used for each analysis, as described in each figure legend. Our C57BL/6J mice were from an in-house colony originally obtained from Jackson Laboratories. Upon receiving the *App^NL-G-F^* mice, they were bred with the in-house C57BL/6J mice for several generations, then maintained as a homozygous colony. However, since the homozygous colonies were not littermate controls, they were not compared across genotypes. Animal use was approved by the University of North Dakota Institutional Animal Care and Use Committee. The animals were provided food and water *ad libitum* and housed with a 12-h light/dark cycle.

### 2.2. Animals and Treatment

**Figure 1:**
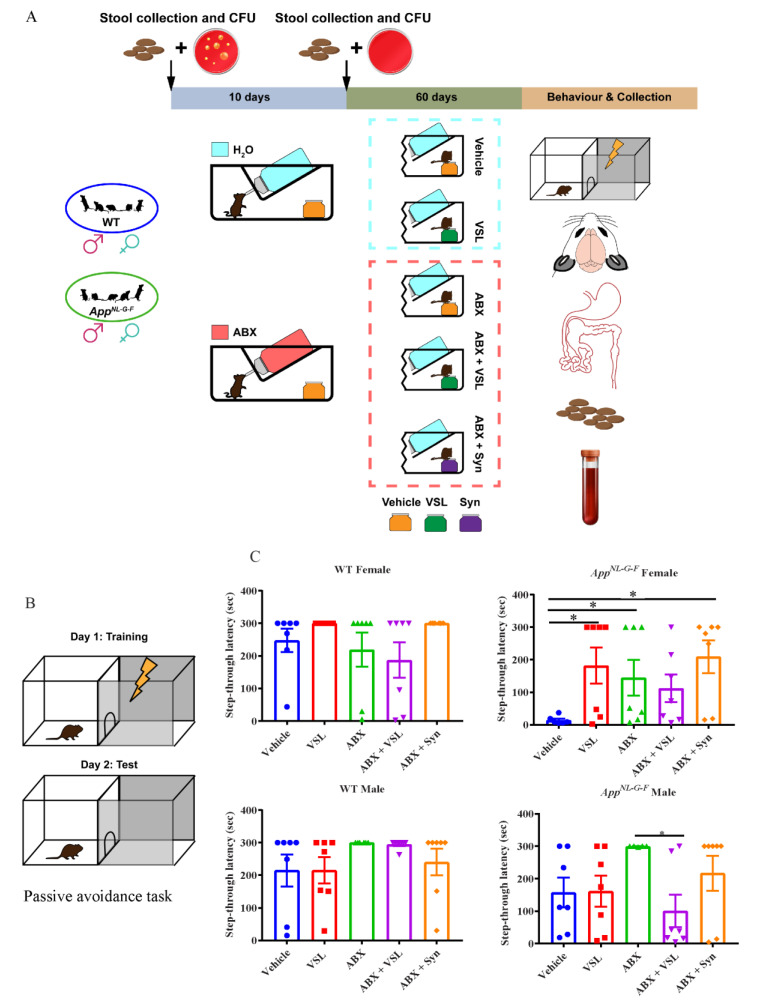
Study design and treatment effects of probiotic and antibiotics on learning and memory. (**A**) General experimental procedure and a timeline of the study. (**B**) Animals at 4–5 months of age were assessed for learning and memory using a passive avoidance task. The image shows the neurobehavior training setup, where a mild foot shock was used as an unconditioned stimulus on day 1 (training day) and the step-through latencies were measured when animals entered from a lit compartment to a dark compartment. (**C**) The passive avoidance task results are shown as step-through latency on day two and are expressed as mean ± SEM. Significant differences were determined by one-way analysis of variance, * *p* < 0.05 (*n* = 7).

Male and female C57BL/6J and *App^NL-G-F^* mice were randomly assigned into five experimental groups as shown in Figure 1A: (1) vehicle (Veh), (2) supplemented with probiotic VSL#3 (VSL#3), (3) treated with antibiotics only (ABX), (4) treated with antibiotics plus probiotic (ABX + VSL#3), and (5) treated with antibiotics plus probiotic and prebiotic (ABX + Syn). Antibiotics treatment was given before the supplementation of prebiotics and probiotics. Animals in the antibiotics group were given an oral non-absorbable antibiotic cocktail treatment (vancomycin 0.5 mg/mL, neomycin 5 mg/mL, and pimaricin: 1.25 µg/mL) in drinking water for ten days, while animals in other groups were given regular water for the same period. After the antibiotics treatment, the animals in the probiotic or prebiotic group were supplemented with VSL#3 or prebiotic in a regular powered diet (Envigo-Harlan Teklad 22/5 rodent diet, 8640). The treatment was given for eight weeks, and the dose of probiotic VSL#3 (4 × 10^9^ CFU/day/25 g mice) was calculated based on the body surface area normalization method from the recommended human dose of VSL#3 [30,31]. According to the manufacturer’s information, human colonization takes place in 2–3 weeks. However, we elected a longer period of 8 weeks based on the known resistance of mice to probiotic colonization [32]. VSL#3^®^ is a commercially available probiotic cocktail (Alfa Sigma USA, Inc. Covington, LA, USA. Lot No. 809158) of eight strains of lactic acid-producing bacteria: *Lactobacillus plantarum*, *Lactobacillus delbrueckii subsp. Bulgaricus*, *Lactobacillus paracasei*, *Lactobacillus acidophilus*, *Bifidobacterium breve*, *Bifidobacterium longum*, *Bifidobacterium infantis*, and *Streptococcus salivarius subsp. Thermophilus*. The prebiotic used in our study, Prebiotin™ prebiotic fiber supplement with oligofructose-enriched-inulin (Jackson GI Medical, Harrisburg, PA, USA), was supplemented at a dose of 1.2 mg/day/20 g mouse body weight in a regular powdered diet. The amount of prebiotic was calculated based on the human dose.

### 2.3. Antibodies and Reagents

Antibiotics including vancomycin (V2002-5G), neomycin (N6386-100G), and pimaricin (P9703-25MG) were purchased from MilliporeSigma (Burlington, MA, USA). FITC-dextran was purchased from MilliporeSigma (Burlington, MA, USA), and ELISA kits for TNF-α, IL-1β, and IL-10 were purchased from R&D Systems (Minneapolis, MN, USA). Antibodies against GFAP and Aβ were purchased from Cell Signaling Technology Inc. (Danvers, MA, USA). The anti-Iba1 antibody was purchased from Wako Chemicals USA, Inc. (Richmond, VA, USA). Elite Vectastain ABC reagents, Vector VIP, biotinylated anti-rabbit, and anti-mouse antibodies were purchased from Vector Laboratories Inc (Burlingame, CA, USA). ZymoBIOMICS DNA Kits D4300 (Zymo, Irvine, CA, USA), A Quantibody^®^ Mouse Cytokine Array (QAM-TH-17; RayBiotech, Inc., Norcross, GA, USA), and blood and chocolate agar plates (Thermo Fisher Scientific, Rockford, IL, USA) were also used. Ghost dye^TM^ Violet 510 was purchased from Tonbo Biosciences (San Diego, CA, USA). The antibodies and the clones used in flow cytometry experiments are detailed in Table 1.

### 2.4. Fecal Sample Collection and Microbiome Analysis

After eight weeks of VSL#3 treatment, each mouse was placed separately in a clean cage for 10–30 min, and fecal pellets were collected in a sterile 1.5 mL Eppendorf tube. More than 90% of the mice excreted a fecal pellet within one minute of being placed into a clean cage. When a mouse did not pass stool within 30 min, the mouse was gently picked up vertically by the tail for 20–30 s until a pellet was excreted and collected. Samples were stored at 4 °C until all samples were collected and later stored at −80 °C until DNA isolation for microbiome analysis. The DNA from stool samples were isolated using a Zymo kit (Zymo Research, Irvine, CA, USA) and resolved on a 1% agarose gel (*w*/*v*) containing SYBR Safe DNA Gel Stain (Invitrogen Co., Carlsbad, CA, USA) and visualized under ultraviolet light to assess the quality of isolated DNA samples. The samples were sent to the Washington University Genome Sequencing Center core facility for microbiome analysis via 16S rRNA sequencing, and the following protocol was used for analysis. Seven PCR amplicons representative of all nine 16S variable regions using the primers indicated in Table 2 were generated using the Fluidigm Access Array System. Reaction mixture components included 10× Fast Start High Fidelity buffer without MgCl_2_, 25 mM MgCl_2_, dimethyl sulfoxide, 10 mM PCR Grade Nucleotide Mix, 0.05 U/µL of 5 U/µL FastStart High Fidelity Enzyme Blend, 20× Access Array Loading Reagent, 1 µL DNA, and molecular grade water. The BioMark HD system from Fluidigm was employed for PCR amplification. Reaction products were indexed with ten unique base pair sequences via seven rounds of PCR to combine each index sequence. Forty-eight sample libraries were constructed via sample pooling and bead purification used for cleaning. Illumina MiSeq sequencer (2 × 150 base pair kit) was used for library sequencing. Amplification and sequencing were performed at the Genome Technology Access Center at the McDonnell Genome Institute at Washington University in St. Louis. Demultiplexed reads from the seven amplicons were analyzed using the Multiple 16S Variable Region Species-Level Identification (MVRSION) pipeline [33] to generate a list of microbial species with their corresponding number of reads for each sample. Default parameters were employed for the MVRSION analysis in conjunction with the Silva 16S database, and data were rarefied to a depth of 18,000 reads. Diversity analysis was run for the following sample groupings: male, female. The taxonomic classification results of MVRSION were post-processed via QIIME for the core set of diversity analyses, including alpha and beta diversity, alpha rarefaction, and group significance.

### 2.5. Passive Avoidance Test for Learning and Memory Assessment

Learning and memory in animals were tested using a passive avoidance task following completion of the treatment paradigm. The step-through passive avoidance test apparatus (Gemini Avoidance System, San Diego Instruments, San Diego, CA, USA) consisted of illuminated and nonilluminated chambers (25 cm × 20 cm × 20 cm) separated by a guillotine door (5 cm × 5 cm). The floor of the illuminated and nonilluminated chambers is composed of 2 mm diameter stainless steel rods spaced 6 mm apart. During the acquisition test session, mice were gently placed in the illuminated compartment, and the guillotine door between the two chambers was opened 10 s later. When mice entered the nonilluminated chamber, the door automatically closed, and an electrical foot shock (0.9 mA, 3 s) was delivered through the stainless-steel rods. The retention trial session was carried out after 24 h, and the mice were again placed in the illuminated chamber. The step-through latency or the time taken to enter the nonilluminated chamber was then measured. If the mouse did not enter the nonilluminated chamber within 300 s, the retention trial was ended, and the step-through latency time was scored using 300 s as the upper limit.

### 2.6. Immunohistochemistry

Following the completion of the 8-week treatment paradigm, mice were euthanized with CO_2_ asphyxiation and intracardially perfused with PBS to harvest the brains. The left brain hemisphere from each animal was fixed in 4% paraformaldehyde and prepared for histologic analysis as described previously [34]. Briefly, paraformaldehyde-fixed tissue was embedded in a 15% gelatin (made in 0.1 M phosphate buffer) matrix and immersed in a 4% paraformaldehyde solution for two days, followed by two changes of 30% sucrose for cryoprotection, two days each. The blocks were then flash-frozen using dry ice/isomethylpentane, and 40 µm serial sections were cut on a Leica sliding microtome and stored at 4 °C in PBS with 0.1% sodium azide until used for immunostaining. Anti-Aβ (1:1000), anti-GFAP (1:1000), and anti-Iba1 (1:750) antibodies were used for immunodetection with biotinylated secondary antibodies (1:2500 dilution; Vector Laboratories) and an avidin/biotin complex (Vector ABC kit). Immunoreactivity was visualized using Vector VIP as the chromogen. The stained sections were mounted onto gelatin-coated slides and coverslipped using VectaMount (Vector Laboratories) following a standard dehydrating procedure through a series of increasing ethanol concentrations and Histo-Clear (National Diagnostics, Atlanta, GA, USA). Images were acquired using a Hamamatsu NanoZoomer 2.0 HT system to quantify immunostaining, as described previously [26]. Optical densities from the temporal cortices from two serial sections per mouse were measured using Adobe Photoshop software (Adobe Inc., San Jose, CA, USA). All sections were immunostained and analyzed simultaneously to minimize variability. The background value in an unstained tissue area for each section was set to zero using the curve tool before quantifying the stained areas. The optical density values from two sections/brain and seven brains per condition were averaged and plotted for Aβ, GFAP, and Iba1 staining. 

### 2.7. Enzyme-Linked Immunosorbent Assay (ELISA) for Cytokines

Brains and colons collected from each animal were used for biochemical as well as histological procedures. Right temporal cortices and colon were collected, flash-frozen in liquid nitrogen, and stored at −80 °C for subsequent use. For cytokines analyses, a portion of the flash-frozen parietal cortex was homogenized in 1X PBS containing CHAPS detergent and protease inhibitors using a Bullet Blender Storm homogenizer 24 (Next Advance, Inc, Troy, NY, USA) at medium speed followed by centrifugation at 13,000 rpm for 10 min to collect supernatants. The levels of proinflammatory cytokines, TNF-α, IL-1β, and IL-6, were measured from the supernatants using commercially available ELISA kits (R&D Systems). Total protein concentrations were assessed using the BCA assay (Pierce Biotechnology, Rockford, IL, USA), and equal amounts of protein were added to ELISA wells. The results are expressed as pg/mL per mg of protein.

### 2.8. Antibody Array Detection of Cytokine Levels Using a Mouse Th1/Th2/Th17 Array

Cytokine levels were measured using a Quantibody Mouse Cytokine Array (QAM-TH-17; RayBiotech, Inc., Norcross, GA, USA), which is a multiplexed sandwich ELISA-based quantitative array platform on a glass slide. Multiple cytokines, including IFN-γ, IL-1β, IL-10, IL-12p70, IL-13, IL-17A, IL-17F, IL-2, IL-21, IL-22, IL-23p19, IL-28A, IL-4, IL-5, IL-6, MIP-3α (CCL20), TGF-β, and TNF-α, were quantitatively detected according to the manufacturer’s instructions. One standard glass slide was divided into 16 wells of identical cytokine antibody arrays. Each antibody, together with the positive controls, was arrayed in quadruplicate. Cytokines were quantified from their respective standard curves. The data were analyzed with software provided by the company, and results are represented as fold-change compared to controls. 

### 2.9. Spleen Immune Cell Phenotyping by Flow Cytometry

Spleens were removed from male and female C57BL/6J and *App^NL-G-F^* mice randomly assigned into the five experimental groups described above. Single-cell splenic suspensions were prepared by mashing the spleen using the plunger end of a 1 mL syringe through a 70 μm strainer (ThermoFisher, Waltham, MA, USA) and were collected into 5 mL of Ca/Mg free PBS containing 2% fetal calf serum (HyClone, Logan, UT, USA) (FACS buffer). Cells were washed once, and red blood cells were lysed in RBC lysis buffer (BioLegend, San Diego, CA, USA) for 5 min at room temperature. Single cells were stained for 30 min at 4 °C with a fixable viability dye, Ghost Dye Violet 510 (Tonbo Biosciences, San Diego, CA, USA), to distinguish live from dead cells and ensure proper gating and analysis of viable cells. Cells were pre-incubated for 10 min on ice with a purified recombinant anti-CD16/32 antibody dilution to block Fc receptors. (Fc block, BioLegend, San Diego, CA, USA). After blocking, splenocytes were divided into panels for staining with a cocktail of fluorochrome-conjugated antibodies to characterize surface and/or intracellular markers on (a) monocytes/macrophages (PE-CD11b, PECy7-CD14, BV421-F4/80, APC-CD36, and APC Fire750-Ly6C); (b) naïve and activated T cells (FITC-CD3, BV605-CD4, PE-CD25, BV421-CD28, PECy7-CD62L, and APC-CD69); and (c) Tregs (FITC-CD3, BV605-CD4, APC-CD25, and PE-FoxP3). PerCPCy5-CD45 was added to each panel for leukocyte gating. For FoxP3 staining, we used the FoxP3 Transcription Factor Staining Buffer Set (Thermo Fisher), following the manufacturer’s instructions, after surface staining was completed. Cells were stained in a volume of 50 µL and incubated at 4 °C overnight. A Symphony A3 (BD Biosciences, San Jose, CA, USA) flow cytometer was used to collect a minimum of 20,000 total events. Single stained and fluorescence minus one (FMO) control for every fluorochrome used in the experiment were included to fine-tune compensation and facilitate gating, respectively. The source of the antibodies and the clones used in the study are detailed in Table 1. Data were analyzed using FlowJo V 10.7.1 (BD) after eliminating cell doublets and debris by gating on FSC-H vs. FSC-A and FSA-A vs. SSC-A, respectively. Total live CD45^+^ leukocytes were identified by excluding the cells stained with the Live/Dead fixable viability dye. Dimensionality reduced visualization of *t*-distributed stochastic neighbor embedding (t-SNE) was run using FlowJo V 10.7.1 software. Samples were downsampled to 4960–5000 events, and analysis was run on equal numbers of events per sample. We determined the downsampling number based on the sample with the fewest events acquired. Samples from each group were concatenated into a single file for tSNE analysis. tSNE plots were analyzed with different perplexities to reach an output of visually distinct populations in the spleen with the following parameters (iterations = 5000, perplexity = 30). Cell subsets were identified, manually gated, and overlaid onto tSNE plots and assigned different colors. 

### 2.10. Correlational Analysis

A Pearson’s correlation analysis was performed to understand the association between gut microbiota or changes in the bacterial composition and the brain changes observed in animals with antibiotics and VSL#3 treatments. Correlation values greater than 95% were calculated by the MATLAB (version R2017b) and are shown with an asterisk. After correlation, the FDR correction was used to calculate adjusted *p*-values and significant results are presented.

### 2.11. Statistical Analyses

Statistical analyses were performed using Graphpad Prism (Prism version 7.00 for Windows, GraphPad Software, La Jolla, CA, USA). Strains and genotypes were compared separately within their five treated groups. Outliers and normality were assessed on each dataset, except the behavior data, using the Grubb’s and Shapiro–Wilk tests, respectively. If the data followed a normal distribution, one-way ANOVA multiple comparisons with a post-hoc Tukey’s honesty significant difference (HSD) was performed. However, in datasets that were not normally distributed, a non-parametric test with post-hoc Dunn’s test was performed. All plots were generated using GraphPad software. An unpaired *t*-test was used to compare the basal genus differences in male and female C57BL/6 wild type and male and female *App^NL-G-F^* mice without any treatment. Results were presented as mean values ± the standard error of the mean (SEM). Differences were considered significant when *p* < 0.05 and indicated in the figure legend as appropriate.

## 3. Results

### 3.1. Antibiotics and Probiotic Treatment Had Sex-Dependent Effects on Learning and Memory in Wild Type and App^NL-G-F^ Mice

Since our prior work has shown behavioral impairment in this line as early as 3 months of age [35], we evaluated the effect of probiotic and antibiotics treatments on learning and memory using a passive avoidance task following completion of the treatment paradigm (Figure 1B). The step-through latency, or the time taken by an animal to enter from a lit compartment to a dark compartment, was measured. Vehicle-treated *App^NL-G-F^* female mice had decreased step-through latency, which was increased following antibiotics, VSL#3, or antibiotics + synbiotic treatments, demonstrating beneficial effects of both VSL#3 and antibiotics treatments in these mice (Figure 1C). On the other hand, vehicle-treated *App^NL-G-F^* male mice showed a higher basal step-through latency with no additional improvements with any of the treatments indicating overall better memory performance than female mice regardless of the interventions (Figure 1C). Neither male nor female C57BL/6J wild type mice had differences in performance across the treatment groups (Figure 1C). 

### 3.2. Antibiotics and Probiotic Treatments Had Sex-Dependent Effects on Aβ Plaque Load in App^NL-G-F^ Mice

**Figure 2:**
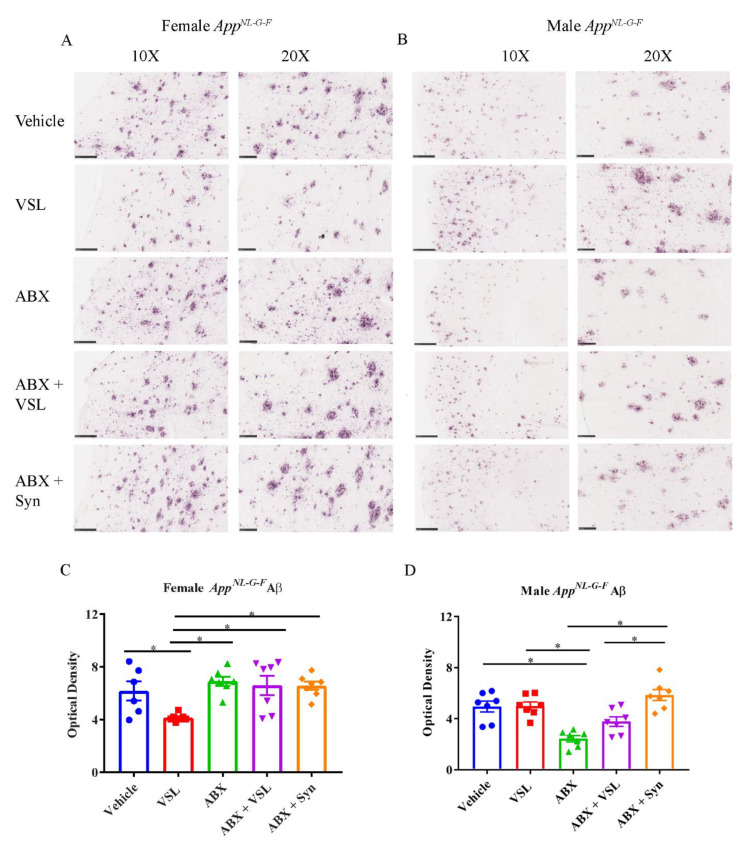
Effect of probiotic and antibiotics treatments on Aβ accumulation in *App^NL-G-F^* brains. Representative immunohistochemical staining images for Aβ from temporal cortices of mice treated with vehicle, VSL#3, antibiotics (ABX), antibiotics+VSL#3 (ABX + VSL), and antibiotics+VSL#3+prebiotic (ABX + Syn) are shown for (**A**) female and (**B**) male *App^NL-G-F^* mice. (**C**,**D**) Quantitation of immunostaining was performed from two sections from each animal, and optical density values were averaged and presented as mean ± SEM. Significant differences were determined by a one-way analysis of variance, * *p* < 0.05 (*n* = 7). Scale bars are 100 µm (10×) and 50 µm (20×).

Since both VSL#3 and antibiotics treatment improved memory in *App^NL-G-F^* female mice, we further assessed whether these treatments also affected the Aβ plaque load in their brains. Therefore, we performed immunohistochemistry for Aβ on serial brain sections from male and female *App^NL-G-F^* mice following the treatment. As expected, vehicle-treated *App^NL-G-F^* mice had robust brain Aβ deposition. However, VSL#3 treatment reduced Aβ plaque immunoreactivity only in female *App^NL-G-F^* mice with no effects in males compared to their respective vehicle-treated groups (Figure 2A–D). On the other hand, antibiotics treatment of *App^NL-G-F^* male mice produced a significant reduction in Aβ immunoreactivity compared to vehicle controls (Figure 2B). These results demonstrated sex-dependent differential effects of antibiotics and probiotic treatments in which probiotics attenuated plaque load in females and antibiotics in males. 

### 3.3. Antibiotics and Probiotic Treatment Had Sex- and Strain-Dependent Effects on Gliosis in Wild Type and App^NL-G-F^ Mice

**Figure 3:**
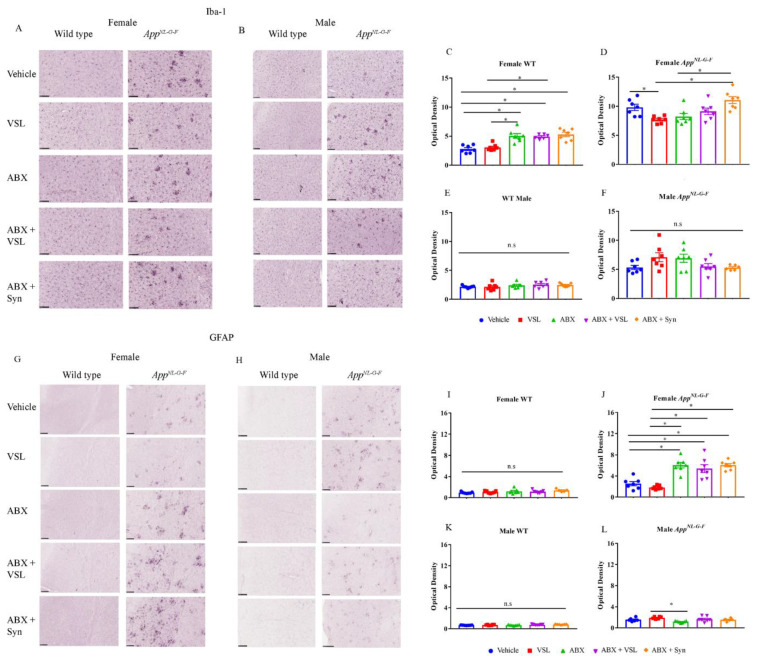
Effect of probiotic and antibiotics treatments on Iba1 and GFAP immunoreactivity. Representative immunohistochemical staining images for (**A**,**B**) Iba1 and (**G**,**H**) GFAP from temporal cortices of female and male WT and *App^NL-G-F^* mice treated with vehicle, VSL#3, antibiotics (ABX), antibiotics+VSL#3 (ABX + VSL), and antibiotics+VSL#3+prebiotic (ABX+Syn) are shown. Quantitation of (**C**–**F**) Iba1 and (**I**–**L**) GFAP immunostaining was performed from two sections from each animal, and optical density values were averaged and presented as mean ± SEM. Significant differences were determined by a one-way analysis of variance, * *p* < 0.05 (*n* = 7). Scale bars are 50 µm (20×).

Since Aβ levels were reduced following VSL#3 supplementation and antibiotics treatment in females and males, respectively, we examined whether the associated gliosis would also be affected. We again performed immunohistochemistry using serial brain sections from the C57BL/6J and *App^NL-G-F^* mice. VSL#3 feeding decreased Iba1 immunoreactivity compared to vehicle controls in *App^NL-G-F^* females with no effects on *App^NL-G-F^* males or wild type mice (Figure 3A–F). Antibiotics treatment had no impact on Iba1 immunoreactivity in either *App^NL-G-F^* male or female mice compared to vehicle controls (Figure 3A–F). Interestingly, wild type females, but not males, treated with antibiotics in all three groups, antibiotics, antibiotics + VSL#3, and antibiotics + synbiotic, showed significant increases in Iba1 immunoreactivity compared to vehicle controls indicating exacerbation of microgliosis following antibiotics treatment (Figure 3A–F).

We next assessed astrogliosis in the treatment groups using an anti-GFAP antibody to stain serial brain sections. VSL#3 treatment had no significant effects on male or female C57BL/6J or *App^NL-G-F^* immunoreactivity (Figure 3G–L). On the other hand, antibiotics treatment resulted in a marked increase in GFAP immunoreactivity in all the antibiotics-treated groups in *App^NL-G-F^* females compared to vehicle controls, suggesting increased astrocyte activation (Figure 3G–L). 

### 3.4. Antibiotics and Probiotic Treatment Had Sex-Dependent Effects on Brain Cytokines in Wild Type and App^NL-G-F^ Mice

**Figure 4:**
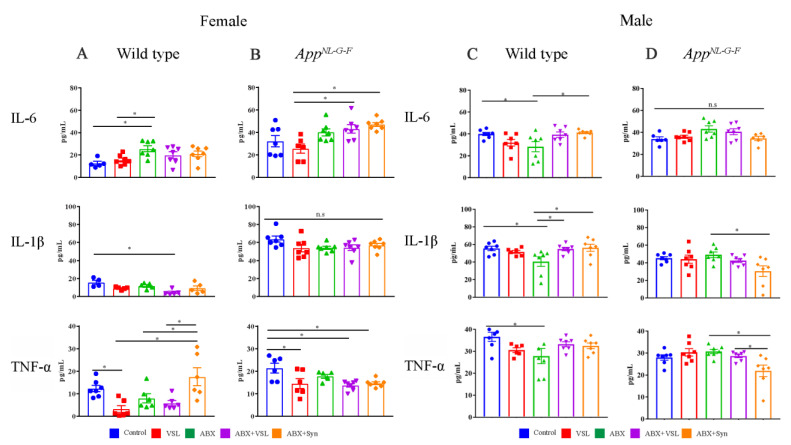
Effect of probiotic and antibiotics treatments on proinflammatory cytokines in female and male mice. Protein levels of interleukin-6 (IL-6), IL-1β, and tumor necrosis factor (TNF-α) were quantified from lysates prepared from parietal cortices of WT (**A**) and *App^NL-G-F^* (**B**) female mice and WT (**C**) and *App^NL-G-F^* (**D**) male mice treated with vehicle, VSL#3, antibiotics (ABX), antibiotics+VSL#3 (ABX + VSL), and antibiotics+VSL#3+prebiotic (ABX + Syn). Lysates were analyzed by enzyme-linked immunosorbent assay (ELISA). Data are presented as mean ± SEM. Significant differences were determined by one-way analysis of variance, * *p* < 0.05 (*n* = 7).

Since glial immunoreactivity changes were observed in female *App^NL-G-F^* brains following treatments, we assessed select brain cytokine concentrations next. Levels of the proinflammatory cytokines IL-6, IL-1β, and TNF-α, were quantified from parietal cortex lysates. VSL#3 supplementation decreased TNF-α levels in both wild type and *App^NL-G-F^* females compared to vehicle controls with no significant effect on the other cytokines (Figure 4A,B). In fact, treatments with probiotic including VSL#3 alone, antibiotics + VSL#3, and antibiotics + synbiotic all decreased TNF-α levels compared to vehicle controls in *App^NL-G-F^* female mice (Figure 4A,B). Interestingly, antibiotics treatment increased IL-6 levels in wild type female mice compared to vehicle controls (Figure 4A). In contrast, concentrations of all three cytokines were decreased in antibiotics-treated wild type males compared to vehicle controls. (Figure 4C). In addition, brain cytokine levels were not altered by any treatment in *App^NL-G-F^* males compared to their vehicle controls (Figure 4D). These results also provide convincing evidence that treatments with probiotics and antibiotics had sex- and strain-specific effects on the brain.

### 3.5. Antibiotics and Probiotic Treatment Had Sex-Dependent Effects on the Intestinal Microbiome of Wild Type and App^NL-G-F^ Mice

Based on the differential effects of treatments on male and female mice, we hypothesized that this was due to sex differences in the basal intestinal microbiome or microbiome response to the treatments. Therefore, the composition of fecal bacterial communities in wild type and *App^NL-G-F^* mice was examined using 16S rRNA sequencing following the treatments. Interestingly, several genera were more abundant in males compared to females in both wild type and *App^NL-G-F^* mice. Specifically, we found an increase in *Enterohabdus*, *Prevotella*, *Alistipes*, *Parabacteroides*, *Mucispirillium*, *Streptococcus*, *Intestinimonas*, *Candidatus Arthromitus*, *Clostridium*, *Anaerostipes*, *Oscillibacter*, *Ruminiclostrodium*, *Parasutterella*, and *Helicobacter* in wild type males when compared to wild type females (Figure S1). Similarly, increases in *Prevotella*, *Alistipes*, *Parabacteroides*, *Clostridium*, *Adlercrutzia*, *Bacteroides*, and *Akkermansia* were observed in *App^NL-G-F^* males compared to *App^NL-G-F^* females (Figure S1). In contrast, *Lactobacillus* was decreased in both wild type and *App^NL-G-F^* males compared to females. In addition, there were several common genera in low or high abundance based on a male or female comparison in both wild type and *App^NL-G-F^* mice (Figure S1). These results made it clear that sex differences in microbiota composition exist in both wild type and *App^NL-G-F^* mice, which may contribute to sex-specific effects of VSL#3 and antibiotics in male and female mice.

**Figure 5:**
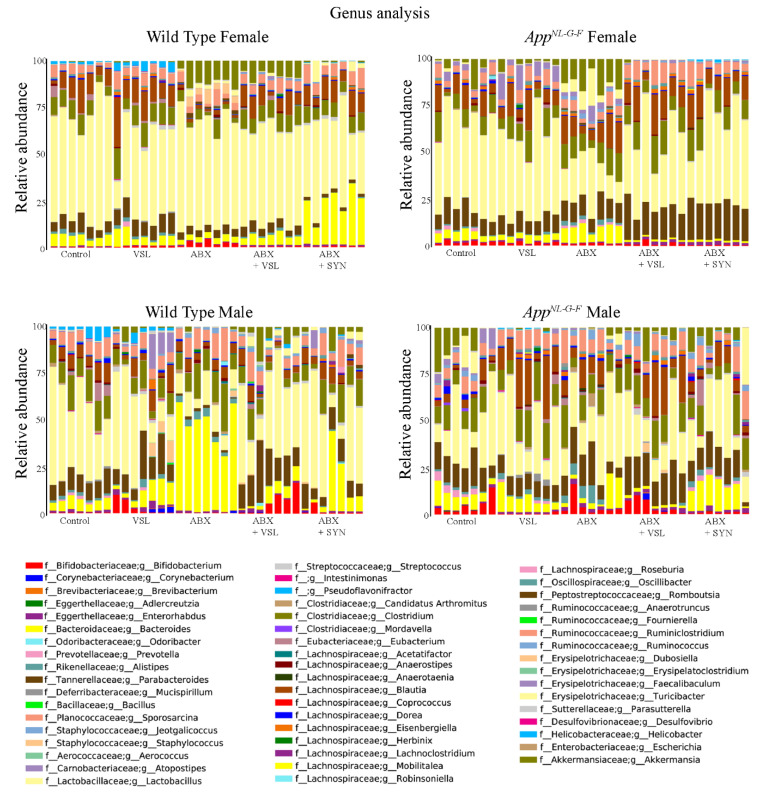
Effects of probiotic and antibiotics treatments on gut bacterial diversity in female and male mice. The relative abundance (%) of dominant bacterial genera in fecal samples of C57BL/6 (WT) and *App^NL-G-F^* mice treated with vehicle, VSL#3, antibiotics (ABX), antibiotics+VSL#3 (ABX + VSL), and antibiotics+VSL#3+prebiotic (ABX + Syn) are plotted as bar charts. The relative abundances are based on the proportional frequencies of the DNA sequences classified at the genus level. Seven animals per group were examined. Thus, each color represents a different bacterial genus.

After defining the basal sex differences, we assessed how the various treatment affected the microbiota composition. At the genus level, VSL#3 treatment of wild type females resulted in the increased relative abundance of several bacterial taxa, including *Parabacteroides*, *Streptococcus*, *Clostridium*, *Oscillibacter*, *Helicobacter*, *Mucispirillum*, *Intestinimonas*, and *Desulfovibrio* and a decrease in abundance of *Lactobacillus* compared to vehicle-treated animals (Figure 5). On the other hand, antibiotics treatment of wild type females increased the abundance of *Bifidobacterium*, *Romboutsia*, *Dubosiella*, *Parasutterella*, *Akkermansia*, and *Turicibacter* and decreased *Blautia*, *Candidatus Arthromitus*, *Alistipes*, *Clostridium*, *Parabacteroides*, *Dorea* and *Oscillibacter* compared to vehicle-treated mice. Antibiotics + VSL#3 supplementation in wild-type females increased the abundance of Streptococcus, Enterorhabdus, Ruminococcus, and Akkermansia and decreased Parabacteroides and Lactobacillus compared to those in wild-type females to vehicle-treated mice. Antibiotics + synbiotics supplementation in these animals increased *Bacteroides*, *Streptococcus*, *Enterorhabdus*, and *Ruminiclostridium* and decreased *Lactobacillus*, *Allistipes*, *Parabacteroides*, and *Eubacterium* compared to vehicle controls (Figure 5). *App^NL-G-F^* female mice supplemented with VSL#3 had increased abundance of *Streptococcus*, *Faecalibaculum*, *Anaerotruncus*, and *Parasutterella* and decreased *Candidatus Arthromitus*, *Prevotella*, and *Dorea* when compared to their vehicle controls. In contrast, antibiotics treatment of *App^NL-G-F^* females increased the abundance of *Bacteroides*, *Alistipes*, *Turicibacter*, *Ruminococcus*, *Romboutsia*, *Anaerotruncus*, and *Akkermansia* and decreased abundance of *Candidatus Arthromitus*, *Lactobacillus*, and *Dorea* compared to vehicle controls (Figure 5). Antibiotics + VSL#3 treatment increased *Streptococcus*, *Enterorhabdus*, *Parabacteroides*, and *Ruminiclostridium* and decreased *Bacteroides*, *Erysipelatoclostridium*, and *Alistipes* when compared to vehicles. The antibiotics + synbiotic-treated group of female mice showed increased abundance of *Streptococcus*, *Enterorhabdus*, *Parabacteroides*, and *Helicobacter* and decreased *Bifidobacterium*, *Bacteroides*, *Dorea*, and *Alistipes* abundance compared to vehicle-treated mice (Figure 5). 

In wild type male mice, treatment with VSL#3 increased abundance of *Corynebacterium*, *Brevibacterium*, *Streptococcus*, *Staphylococcus*, and *Faecalibaculum* and decreased abundance of genera such as *Prevotella* compared to the vehicle group. Antibiotics treatment of WT males increased *Bacteroides*, *Allistepes*, and *Ruminococcus* and decreased *Pervotella*, *Lactobacillus*, *Parabacteroides*, *Helicobacter*, and *Intestinimonas* compared to vehicle mice (Figure 5). Antibiotics + VSL#3 treatment increased *Bifidobacterium*, *Ruminicoccus*, *Erysipelatoclostridium*, *Turicibacter*, *Parasutterella*, and *Akkermansia* and decreased *Prevotella*, *Helicobacter*, *Mucispirillum*, and *Intestinimonas*. Wild type male mice treated with the antibiotics + synbiotic showed increased abundance of *Streptococcus* and *Ruminocccus* and decreased abundance of *Mucispirillum*, *Intestinimonas*, and *Helicobacter.* In contrast, *App^NL-G-F^* males treated with VSL#3 had increased *Mucispirillium*, *Blautia*, *Intestinimonas*, and *Helicobacter* abundance and decreased *Prevotella*, *Aldlercreutzia*, *Faecalibaculum*, and *Akkermansia* abundance when compared to vehicle-treated males. Antibiotics treatment of *App^NL-G-F^* males resulted in increased abundance of *Candidatus Arthromitus*, *Alistipes*, and *Erysipelatoclostridium* and decreased *Prevotella*, *Faecalibaculum*, and *Akkermansia* abundance compared to vehicles. Antibiotics + VSL#3 treatment increased abundance of *Enterorhabdus* and *Blautia* and decreased abundance of *Aldlercreutzia*, *Prevotella*, and *Fecalibaculum*, when compared to vehicle-treated *App^NL-G-F^* males. Antibiotics + synbiotic feeding increased the abundance of *Bacteroides* and *streptococcus* and decreased *Prevotella* abundance compared to vehicle males (Figure 5). 

### 3.6. Specific Gut Bacterial Compositions Were Associated with Brain Changes in C57BL/6J and App^NL-G-F^ Mice 

**Figure 6:**
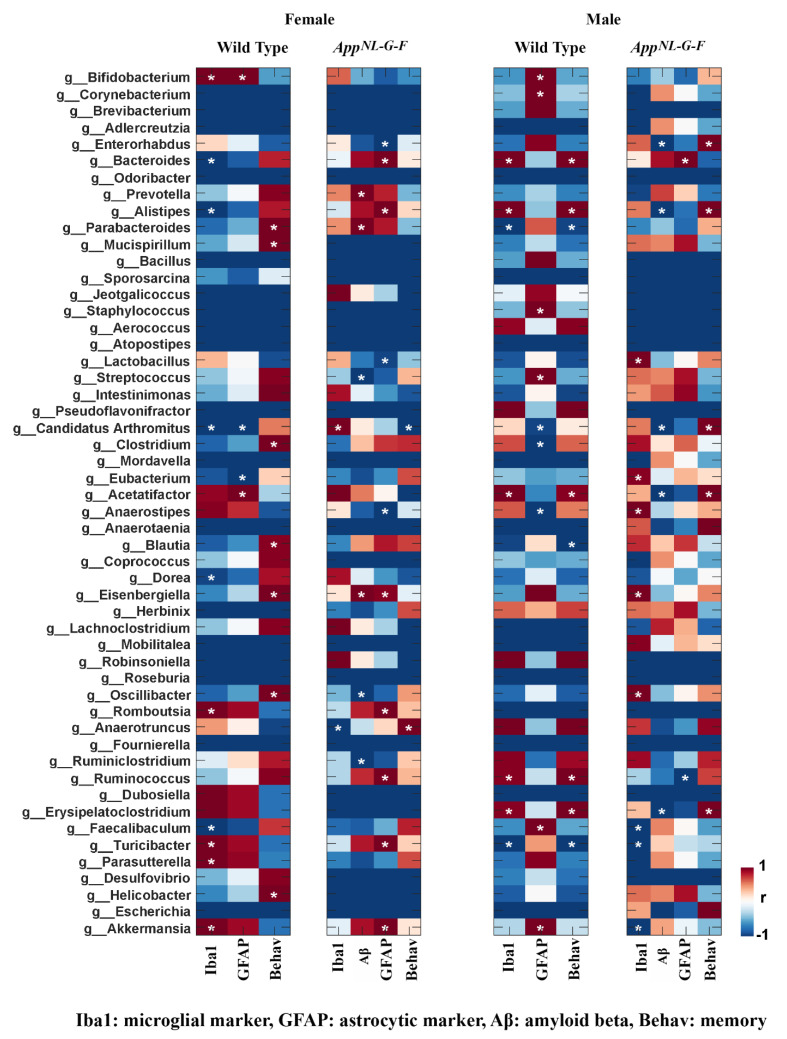
Correlation of Aβ, behavior, and gliosis with fecal microbiome genera in female and male mice. Heatmaps show the Pearson’s correlation between specific gut genera and brain changes observed in control, probiotic-, and antibiotics-treated groups. Blue to red are r values ranging from negative 1 to positive 1: red color, positive correlation; blue color, negative correlation. The asterisk indicates a significant correlation (more than 95%) between genus and brain data.

Following treatments, we explored the relationship between gut microbiota and brain changes by performing Pearson’s correlational analysis between microbiota composition and brain changes, including behavior, Aβ plaque load, and Iba1/GFAP immunoreactivity. The results showed significant correlations between brain changes and bacterial genera (*p* < 0.05). In wild type females, a significant positive correlation existed between Iba1 immunoreactivity and genera, including *Akkermansia*, *Parasutterella*, *Turicibacter*, *Romboutsia*, and *Bifidobacterium*, while a negative correlation was observed with the genera *Feacalibaculum*, *Dorea*, *Candidatus Arthromitus*, *Alistipes*, and *Bacteroides* (Figure 6). Astrocyte GFAP immunoreactivity positively correlated with the genera *Acetatifacter* and *Bifidobacterium* and negatively correlated with the genera *Candidatus*, Arthromitus, and *Eubacterium* (Figure 6). In female *App^NL-G-F^* mice, the genus *Anaerotruncus* was negatively correlated, and the genus *Candidatus* Arthromitus was positively correlated with brain Iba1 immunoreactivity (Figure 6). Interestingly, the same genera correlated, but in a reverse fashion, with the learning and memory associated behavioral changes in *App^NL-G-F^* females (Figure 6). Aβ immunoreactivity in *App^NL-G-F^* females positively correlated with *Prevotella*, *Parabacteroides*, and *Eisenbergiella* and negatively correlated with *Ruminiclostridium*, *Oscillibacter*, and *Streptococcus* (Figure 6). GFAP immunoreactivity positively correlated with *Akkermansia*, *Turicibacter*, *Ruminococcus*, *Romboutsia*, *Eisenbergiella*, *Allistipes*, and *Bacteroides* and negatively correlated with *Anaerostipes*, *Lactobacillus,* and *Enterorhabdus* (Figure 6). 

Wild type males had positive correlations of Iba1 immunoreactivity and behavior with genera, including *Erysipelatoclostridium*, *Ruminicoccus*, *Acetatifacter*, *Alistipes*, and *Bacteroides* and a negative correlation with the genera *Turicibacter* and *Parabacteroides*. In wild type males, a positive correlation was found between GFAP immunoreactivity and the genera *Bifidobacterium*, *Corynebacterium*, *Staphylococcus*, *Streptococcus*, *Fecalibaculum*, and *Akkermansia* and a negative correlation with the genera *Candidatus* Arthromitus, *Anaerostipes*, and *Clostridium* (Figure 6). In *App^NL-G-F^* males, Iba1 immunoreactivity positively correlated with the bacterial genera *Oscillibacter*, *Eisenbergiella*, *Anaerostipes*, *Eubacterium*, and *Lactobacillus* and negatively correlated with the genera *Akkermansia*, *Turicibacter*, and *Faecalibaculum* (Figure 6). GFAP immunoreactivity in *App^NL-G-F^* males negatively correlated with *Ruminococcus* and positively correlated with *Bacteroides*. Interestingly, genera including *Erysipelatoclostridium*, *Acetatifacter*, *Candidatus* Arthromitus, *Alistipes*, and *Enterorhabdus* negatively correlated with Aβ immunoreactivity and positively correlated with behavior in *App^NL-G-F^* males (Figure 6). 

### 3.7. Gut Microbiota Alteration Had Sex-Specific Effects on Intestinal Permeability and Colon Cytokine Levels

An imbalance in gut microbiota, or dysbiosis, is known to affect intestinal permeability. Since we observed differences in gut microbiota composition in wild type and *App^NL-G-F^* mice with and without treatments, we further investigated whether basal state or treatment-induced differences in intestinal leakiness were present in the mice. An in vivo FITC-dextran assay was performed in which FITC-conjugated dextran (4 kDa) was administered orally to animals, and intestinal permeability was assessed by quantifying the level of fluorescence in their serum samples. There was no difference in intestinal permeability across any group of wild type or *App^NL-G-F^* female mice (Figure S2). However, we observed a significantly higher amount of FITC-dextran in the serum of the antibiotics + VSL#3-treated group in wild type males, suggesting compromised intestinal permeability in this group compared to controls (Figure S2). A similar increase in intestinal permeability was observed in the antibiotics + synbiotic treatment group of *App^NL-G-F^* males (Figure S2). 

**Figure 7:**
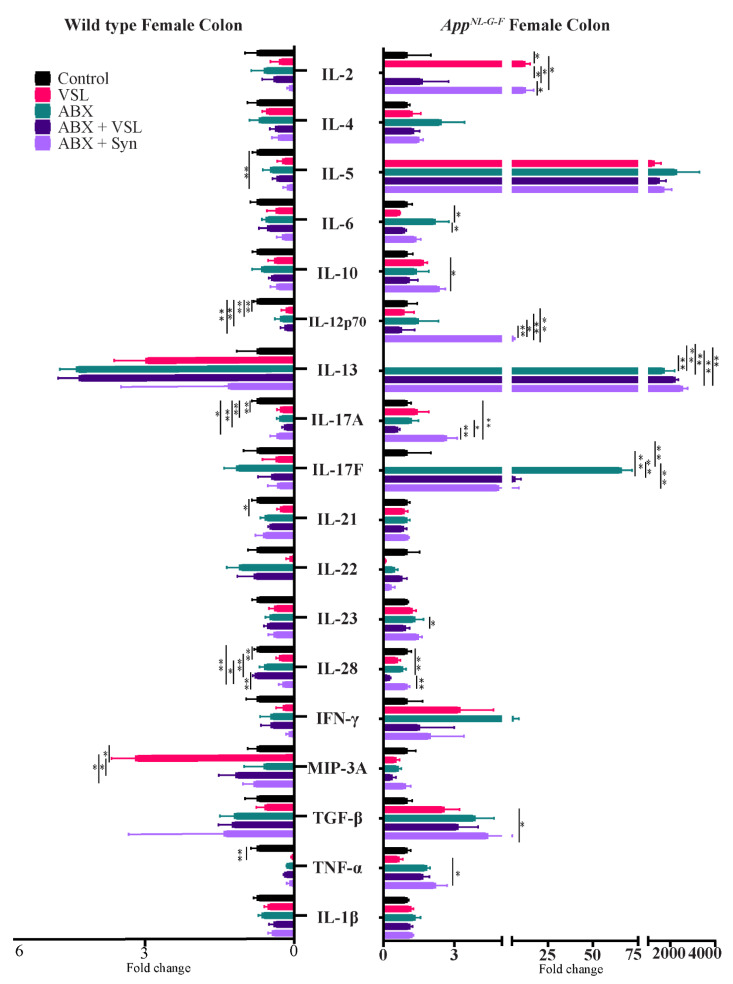
Quantification of colon levels of Th1, Th2, and Th17 cytokines in female C57BL/6J (WT) and *App^NL-G-F^* mice. Colons were lysed from vehicle, VSL#3, antibiotics (ABX), antibiotics+VSL#3 (ABX + VSL), and antibiotics+VSL#3+prebiotic (ABX + Syn) female WT mice to quantify cytokine levels via commercial slide array. Data are presented as fold change with respect to controls (*n* = 5). If the controls values were zero, we used 0.1 as an arbitrary value for that group to calculate the fold change in that dataset. Significant differences were determined by a one-way analysis of variance, * *p* < 0.05, ** *p* < 0.01.

**Figure 8:**
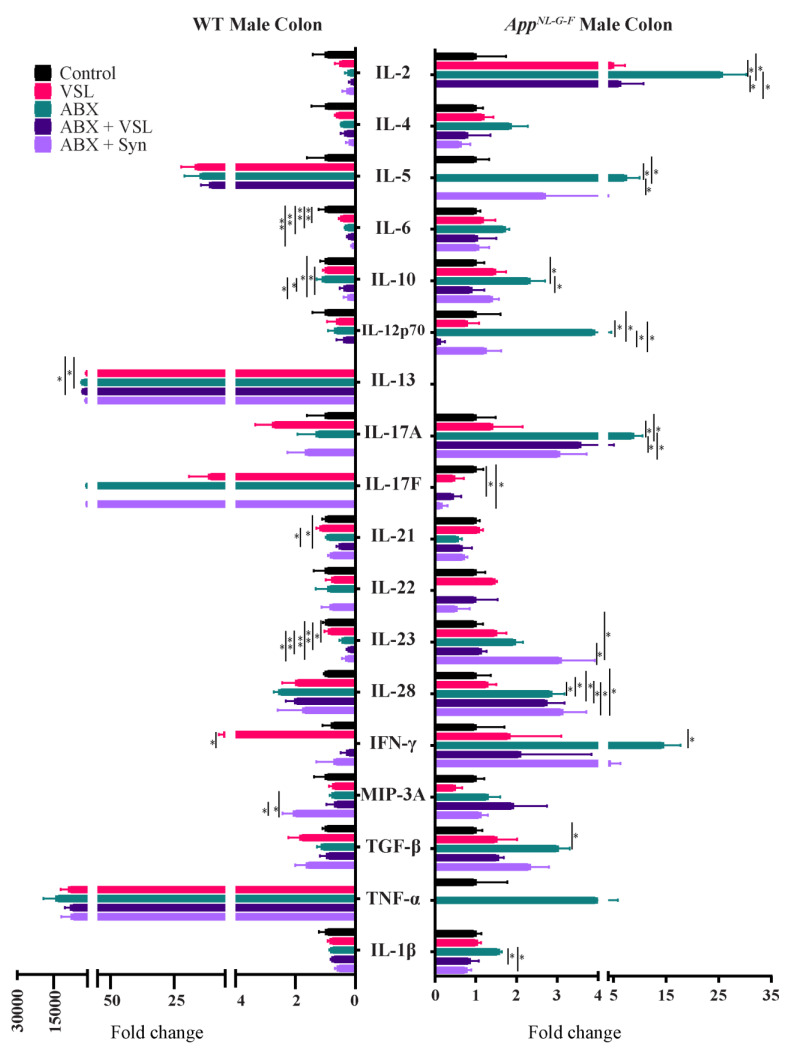
Quantification of colon levels of Th1, Th2, and Th17 cytokines in male C57BL/6J (WT) and *App^NL-G-F^* mice. Colons were lysed from vehicle, VSL#3, antibiotics (ABX), antibiotics+VSL#3 (ABX+VSL), and antibiotics+VSL#3+prebiotic (ABX + Syn) male WT mice to quantify cytokine levels via commercial slide array. Data are presented as fold change with respect to controls (*n* = 5). If the controls values were zero, we used 0.1 as an arbitrary value for that group to calculate the fold change in that dataset. Significant differences were determined by a one-way analysis of variance, * *p* < 0.05, ** *p* < 0.01.

Since the gut microbiota is well known to modulate host immune response and alter intestinal and circulating levels of cytokines [36,37], we next assessed changes in the intestinal cytokine levels across treatments despite lack of differences in gut leakiness. The levels of several Th1, Th2, and Th17 cytokines were measured in colon tissue using a cytokine array. Wild type females had decreased TNF-α, IL-28A, IL-21, IL-17A, and IL-12p70 levels and an increase in MIP-3α following VSL#3 supplementation compared to vehicle-treated mice (Figure 7). Antibiotics, antibiotics + VSL#3, and the antibiotics + synbiotic groups in wild type females also had decreased IL-12p70 and IL-17A levels compared to controls (Figure 7). In addition, IL-28A and IL-5 concentrations were decreased in the antibiotics + synbiotic groups compared to vehicle wild type females (Figure 7). On the other hand, *App^NL-G-F^* females had increased IL-2 levels following VSL#3 supplementation and increased IL-6, IL-13 and IL-17F after antibiotic treatment compared to vehicle-treated controls (Figure 7). The antibiotics + synbiotic group of *App^NL-G-F^* females had increased IL-2, IL-10, IL-12 p70, IL-13, L-17A, and TGF-β levels compared to vehicle-treated mice (Figure 7). On the other hand, all treatments decreased IL-6 levels in the wild type males compared to vehicle controls (Figure 8). Antibiotics treatment also decreased IL-23 and increased IL-13 levels in wild type males compared to vehicle controls (Figure 8). VSL#3 treatment did not affect cytokine levels in *App^NL-G-F^* males compared to vehicle controls (Figure 8). However, the antibiotics treatment of *App^NL-G-F^* males stimulated a marked increase in IL-2, IL-5, IL-10, IL-12p70, IL-28A, IFN-γ, TGF-β, and IL-17A levels, and decreased in IL-17F compared to vehicle mice (Figure 8). The antibiotics + synbiotic group of male *App^NL-G-F^* mice had increased IL-28A and IL-23 levels but decreased IL-17F compared to vehicle-treated mice (Figure 8). 

### 3.8. Antibiotics and Probiotic Treatments Altered Peripheral Immune Cell Phenotype in a Sex-and Genotype-Dependent Fashion

To examine whether the microbiome manipulations altered peripheral immune cell phenotype or circulating cytokines as a possible communication mechanism to the brain, we quantified levels of select Th1, Th2, and Th17 cytokines in serum and phenotyped the spleen immune subsets. Wild type females had decreased IL-12p70, IL-17A, and IL-28A in both the VSL#3- and antibiotics-treated groups compared to vehicle controls (Figure S3). Antibiotics treatment also decreased IL-12p70, IL-17A, IL-28, and IL-23 while increasing IL-13 in female wild type compared to vehicle mice (Figure S3). On the other hand, antibiotics treatment of *App^NL-G-F^* females increased serum levels of IL-4, IL-6, IL-10, and IL-12p70 compared to vehicle-treated mice while these cytokines were reduced in the antibiotics + VLS#3-treated group (Figure S3). No effects of VSL#3 on circulating cytokines were observed in *App^NL-G-F^* females (Figure S3). All treatments decreased IL-12p70 levels in wild type male mice compared to vehicle-treated controls (Figure S4). No significant effects of VSL#3 were observed in *App^NL-G-F^* males compared to vehicle-treated mice, although the antibiotics treatment decreased levels of IL-22 and increased IFN-γ in serum (Figure S4).

**Figure 9:**
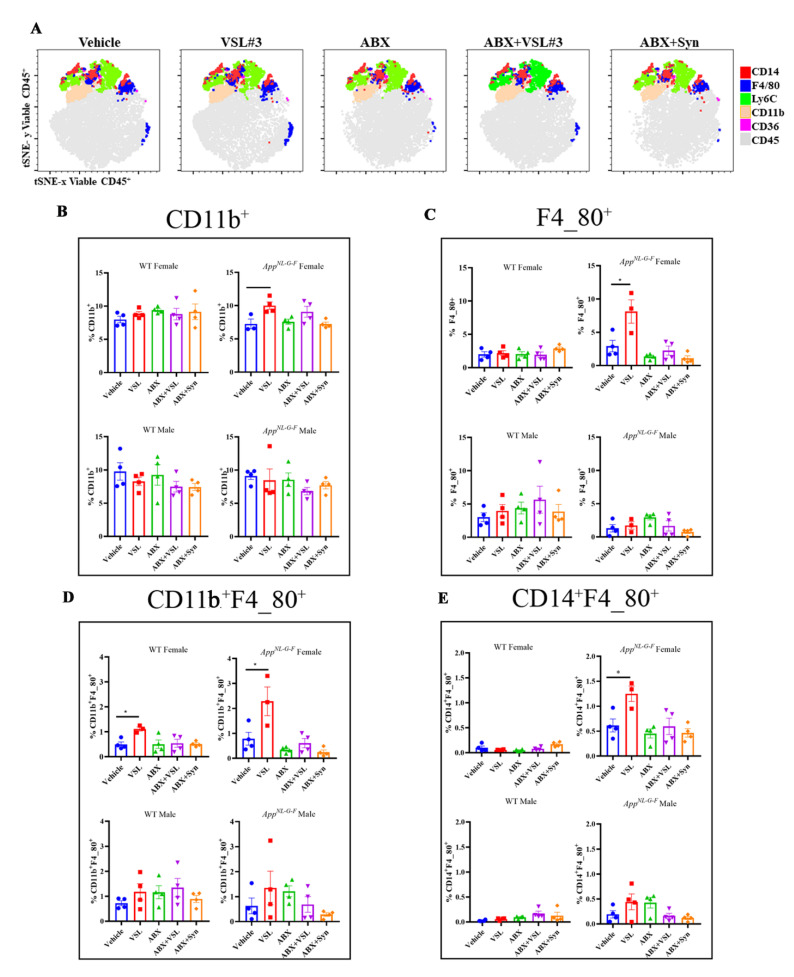
Upregulation of CD11b^+^, F4_80^+^, CD11b^+^F4_80^+^, and CD14^+^F4_80^+^ macrophages in VSL#3-treated female *App ^NL-G-F^* mice. Splenocytes were stained with a panel of cell surface markers and measured by flow cytometry. tSNE analysis was run on 4960–5000 live CD45^+^ single cells per sample using all surface markers, and manually gated populations were overlaid to visualize the subsets. (**A**) The data presented are representative tSNE plots for female *App^NL-G-F^* mice, generated by concatenating individual samples in each treatment group with the following parameters: iterations: 5000 and perplexity: 30. (**B**–**E**) Bar graphs show the mean ± SEM of the percentage of CD11b^+^, F4_80^+^, CD11b^+^F4_80^+^, and CD14^+^F4_80^+^ cells across different treatment groups in female and male C57BL/6 (WT) and *App ^NL-G-F^* mice (*n* = 3–4 mice/group). Statistically significant differences were computed by a one-way ANOVA, * *p* < 0.05, ** *p* < 0.005.

**Figure 10:**
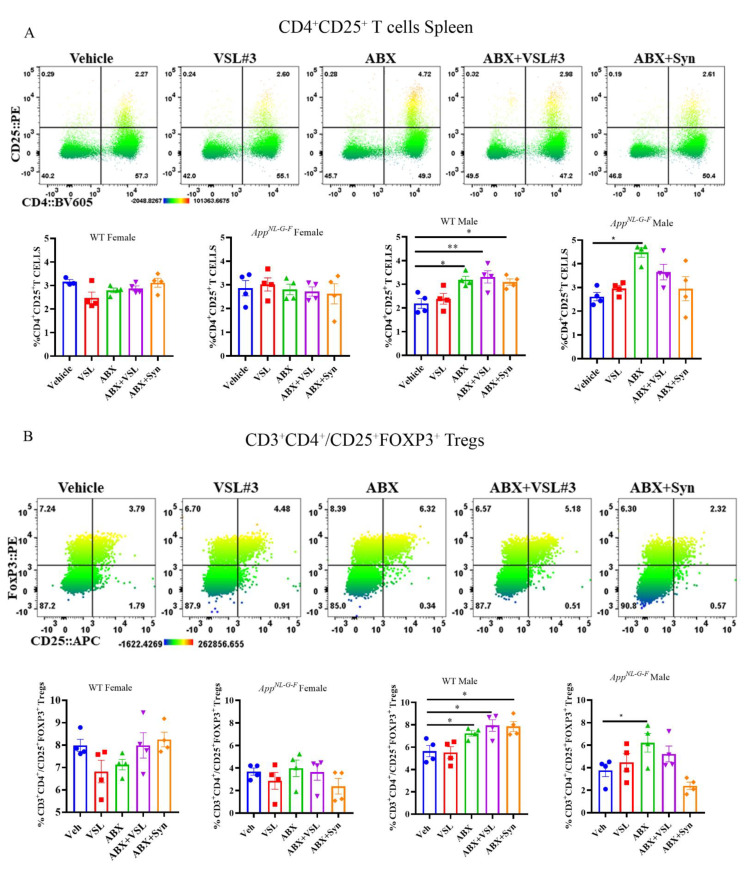
Upregulation of CD4^+^CD25^+^ T and FoxP3 expressing CD4^+^CD25^+^ T regulatory cells following antibiotics treatment in male WT and *App ^NL-G-F^* mice. (**A**) Splenocytes were stained with surface markers directed against a panel of T cell markers and measured by flow cytometry. Representative heat map dot plots from male *App ^NL-G-F^* mice showing the percentage of CD4^+^CD25^+^ T cells gated on viable CD45^+^CD3^+^ cells. Numbers in each quadrant indicate the percentage of cells. The heat map color varies from blue to red, indicating relative under and over-representation of the percentage of cells, respectively. Bar graphs display mean ± SEM of the percentage of CD4^+^CD25^+^ T cell across different treatment groups in female and male C57BL/6 (WT) and *App ^NL-G-F^* mice (*n* = 3–4 mice/group). Statistically significant differences were computed by one-way ANOVA, * *p* < 0.05, ** *p* < 0.005. (**B**) Splenocytes were stained with surface markers directed against a panel of T cell markers and measured by flow cytometry. Representative heat map dot plots from male *App ^NL-G-F^* mice showing the percentage of CD25^+^FoxP3^+^ Tregs gated on viable CD45^+^/CD3^+^CD4^+^ cells. Numbers in each quadrant indicate the percentage of cells. The heat map color varies from blue to red, indicating relative under- and over-representation of the percentage of cells, respectively. Bar graphs display mean ± SEM of percentage of CD25^+^FoxP3^+^ Tregs across different treatment groups in female and male C57BL/6 (WT) and *App ^NL-G-F^* mice (*n* = 3–4 mice/group). Statistically significant differences were computed by one-way ANOVA, * *p* < 0.05, ** *p* < 0.005.

Dissociated splenocytes from the treatment groups were immunophenotyped as an additional measure of monitoring peripheral immune changes due to probiotic, antibiotics, or synbiotic interventions. VSL#3 treatment resulted in a significant increase in CD11b-, F4/80-, and CD14-expressing macrophages in female *App^NL-G-F^* mice compared to their vehicle controls (Figure 9). Although no differences in total CD3 or CD4 lymphocytes were observed across the treatment groups in male or female *App^NL-G-F^* mice, there was a significant increase in CD3^+^/CD4^+^CD25^+^ activated T cells in the antibiotics-treated male group compared to vehicle-treated controls (Figure 10). A similar activation pattern of CD3^+^/CD4^+^CD25^+^ cells was observed in male wild type antibiotic + probiotic treated mice compared to their vehicle controls (Figure 10). We characterized T-regulatory cells (Tregs) based on the co-expression of FoxP3^+^CD25^+^ cells to enable the delineation of immunoregulatory subsets in addition to CD4^+^ CD25^+^ activated/effector T cells. Data presented in Figure 10 suggest that antibiotics treatment increased FoxP3 expressing CD4^+^ CD25^+^ T cells in both WT and *App^NL-G-F^* male mice with no female effects. 

## 4. Discussion

The dietary interventions had different effects on memory performance and plaque load in male versus female mice. For example, there was no effect of any treatment on memory in male *App^NL-G-F^* mice compared to controls. This lack of change was not surprising, since males did not demonstrate a behavioral phenotype compared to wild type mice. Recent studies performed using both male and female *App^NL-G-F^* mice showed greater impairment in social behavior and weaker performance in social olfactory discrimination in females versus males, suggestive of sex differences in behavior impairment in this particular model of AD [38]. It was equally surprising that all treatment groups improved memory in female *App^NL-G-F^* mice. This change suggests that any treatment altering the intestinal microbiome can benefit females, perhaps linking intestinal microbiome changes and memory more tightly in females than males. However, it was compelling that probiotic treatment alone decreased plaque load in female mice, whereas all treatments that involved antibiotics treatment, regardless of subsequent probiotic or synbiotic feeding, did not. Although the mechanism is unclear, this suggests that the neuronal, immune, or metabolic environment caused by VSL#3 feeding facilitates plaque reduction, but an antibiotics-mediated removal of bacteria disrupts intestine biology enough that VSL#3 can no longer provide benefit. Changes in plaque load are not correlative with memory improvement, in general, since behavior improved following probiotic and antibiotics treatments, but plaque load decreased only with probiotic intervention. Human data also indicate that Aβ load does not necessarily correlate with cognitive impairment [39]. 

It was surprising that VSL#3 feeding and antibiotic plus synbiotic treatment did not attenuate plaque load in males, while antibiotics alone did. Perhaps particular bacteria in males are more important for plaque deposition, and their depletion by antibiotics promotes clearance, while synbiotic feeding returns the male intestine to a state of disease-promoting dysbiosis. Our findings are similar to a recent report describing the ability of long-term antibiotics treatment to reduce Aβ deposition and microglial morphological alterations selectively in male but not female APP/PS1 mice [40]. These differential effects of treatments in male and female animals could be due to the high abundance of distinct intestinal bacterial genera, such as *Prevotella*, *Parabacteroides*, *Clostridium*, and *Alistipes*, and low abundance of *Lactobacillus* in males versus females. Another study also showed sex-specific effects of vancomycin and ciprofloxacin-metronidazole in male versus female mice [41]. Our data indicate that although both sexes demonstrated Aβ plaque deposition, female *App^NL-G-F^* mice had a more robust memory deficit at this age compared to males. 

Based on the commonly observed plaque-associated gliosis and inflammatory changes in human AD brains and AD mouse models, we expected glial immunoreactivity and cytokine changes to parallel the differences in plaque load. Instead, we observed a decrease in Iba1 immunoreactivity only in females with probiotic treatment. This correlated with probiotic treatment, which was the only one that decreased amyloid plaque load in females, suggesting that microgliosis in females is related to plaque load. It is unclear why Iba1 immunoreactivity did not reduce in males given antibiotics, even though this reduced plaque load. Perhaps continued microgliosis was occurring due to a role in plaque clearance. Surprisingly, we observed a disconnect between Iba1 and GFAP immunoreactivity changes. For example, all antibiotics treatments increased GFAP immunoreactivity above untreated controls in female *App^NL-G-F^* mice. Thus, it appears that depleting gut bacteria leads to astrogliosis regardless of a probiotic or synbiotic follow-up protocol. The observation that no treatment affected GFAP immunoreactivity in male *App^NL-G-F^* mice indicated that astrocyte and plaque staining are not tightly coupled in males. Previously, it has been reported that the microglial response in human AD brains correlates with Aβ plaque size, whereas the astrocytic response does not [42]. It has been suggested that Aβ serves as a chemotactic signal for activating microglia, and astrocytes primarily respond to plaque-associated neuritic damage [42]. Interestingly, the gut microbiome controls IFNγ secretion from meningeal NK cells to regulate a subset of TRAIL expressing astrocytes, limiting CNS inflammation by inducing T cell apoptosis [43]. Overall, our data indicate that Aβ plaque load and memory performance correlate more with microglial than astrocyte activation in *App^NL-G-F^* females. 

The probiotic treatment was sufficient to reduce brain TNF-α levels in female *App^NL-G-F^* mice, and the reason for a TNF-α selective effect of probiotic treatment observed in females is unclear. Trinitrobenzene sulfonic acid (TNBS) treatment, which is used in animals to induce colitis-like gut inflammation, leads to increased brain TNF-α and reduced memory performance in mice, supporting an association of gut–brain interaction, elevated brain TNF-α, and decreased memory [44]. Although no changes in the levels of male *App^NL-G-F^* brain cytokines were observed with any treatment compared to their respective untreated controls, this was not unexpected, since treatments did not alter GFAP or Iba1 immunoreactivity. However, based on the robust intestinal cytokine changes observed in males following at least antibiotics treatment, it may suggest that gut bacterial changes are not communicating inflammatory events to the brain in males. On the other hand, altering the female intestinal microbiome through probiotic intervention, in particular, was sufficient to decrease brain TNF-α and microglial reactivity, supporting the notion that a gut–brain communication mechanism existed in females.

Consistent with this notion of glial and immune changes corresponding better with behavioral performance compared to plaque load in female mice, we assessed relationships between specific intestinal bacterial genera and brain changes. In *App^NL-G-F^* females, bacterial genera *Anaerotruncus* and *Candidatus* Arthromitus were significantly correlated with both the behavior and Iba1 immunoreactivity in a reverse manner and did not correlate with Aβ levels. Previous reports also showed that probiotics suppress opportunistic pathogens by promoting the growth of *Anaerotruncus*, a genus known to induce Tregs [45,46]. *Candidatus* Arthromitus is a segmented filamentous bacterium that plays a role in regulating intestinal immune functions and is associated with an inflammatory imbalance by eliciting a T helper (Th) 17 immune response in the intestinal lamina propria of mice [47,48]. A decrease of this genus following probiotic and antibiotics treatments in *App^NL-G-F^* females suggests it may potentially affect memory.

On the other hand, the abundance of *Prevotella* and *Eisenbergiella* positively correlated with Aβ levels. Prior work reported a similar increase in AD mice associated with increased severity of cognitive impairment, most likely by disrupting mucosal barrier function and increasing intestinal permeability [49,50]. *Prevotella* is an efficient mucin degrader in the intestine and promotes gastrointestinal dysfunction in diabetes and autism. Furthermore, the increased abundance of *Bacteroides*, *Alistipes*, *Turicibacter*, *Ruminococcus*, *Romboutsia*, and *Akkermansia* positively correlated with increased astrogliosis in female *App^NL-G-F^* mice. Several of these genera have already been described as critical in inflammation and disease [51,52]. For example, *Turicibacter* and *Romboutsia* are short-chain fatty acid-producing bacteria that increased in the antibiotics-treated *App^NL-G-F^* females and correlated with increased GFAP immunoreactivity. These data continue to support the idea that bacterial composition changes, specifically in females, may significantly contribute to a microglial inflammatory phenotype affecting behavior.

In *App^NL-G-F^* males, several genera correlated with Aβ and behavior but in a reverse fashion. Specifically, increased relative abundance of genera including *Erysipelatoclostridium*, *Acetatifacter*, *Candidatus* Arthromitus, *Alistipes*, and *Enterorhabdus* following antibiotics treatment in *App^NL-G-F^* males positively correlated with behavior and negatively correlated with Aβ immunoreactivity. Among these, *Acetatifacter* are acetate and butyrate-producing bacteria, *Enterorhabdus*, which are beneficial for maintaining the mucosal epithelial barrier, and *Alistipes* have protective roles in diseases such as colitis and autism spectrum disorder [53,54]. These data suggest that the use of antibiotics in male *App^NL-G-F^* mice resulted in an enhanced abundance of anti-inflammatory bacterial populations, correlating with the reduced plaque load in their brains. Another recent study using *App^NL-G-F^*, *App^NL-F^*, and wild type mice indicates that the microbiome correlates with behavior, but that the APP genotype in these models modulates this association, possibly by altering the types and number of taxa that reside in the gut [55].

Our assessment of colon and serum cytokine profiles coupled with splenocyte phenotyping confirmed that the male versus female immune response to the microbiome manipulations was quite different. The modest proinflammatory response elicited by probiotic feeding characterized by increased IL-2 and IL-5 levels and increased macrophage/monocytic lineage subsets supported activation of an innate response in the female intestines. Our tSNE-based visualization approach revealed the presence of distinctly unique subsets in VSL#3-treated female *App^NL-G-F^* mice, specifically the macrophage marker, F4_80^+^ cells, that shares surface expression with both CD11b and CD14. The dramatic expansion of these CD11b^+^/F4_80^+^ and CD14^+^F4_80^+^ populations in the VSL#3-treated female *App^NL-G-F^* mice, but not in other treatment groups or male mice, compels further investigations. CD14 is important in the cascade of events involved in recognizing, binding, and cellular responses to bacterial LPS [56]. The increase in CD14 expressing macrophages drives speculation for a correlation between the relative abundance of gram-negative gut microbiota and the induction and expansion of the macrophages following VSL#3 treatment. The endotoxin produced by a diverse gut microbiota may stimulate the influx of splenic macrophage subsets and activate the innate immune system in the periphery and the brain. Although future work needs to define a communication mechanism to the brain, this treatment correlated with the maximum benefits, including improved memory, reduced plaque load and microgliosis, and attenuated brain TNF-α. We have not yet resolved why male *App^NL-G-F^* mice did not respond to the probiotic with similar cytokine and splenocyte changes or why they developed a robust proinflammatory T cell response following antibiotics treatment. 

Nonetheless, it is impressive that perturbation of the gut microbiota with antibiotics treatment conferred an adaptive immune response with increased frequency of CD4^+^CD25^+^ T cells in the male (WT and *App^NL-G-F^*) but not the female mice. The mechanisms underlying the host immunity–microbiome axis is unknown. However, specific metabolites of gut microbes have been reported to maintain the Th17 (IL-17A and IL-17F)/Tregs (TGF-β, IL-10) homeostasis and protective levels of Th1 (IFN-γ) responses [57,58].

Although the antibiotics treatment had few benefits in males or females, the decrease in male plaque load suggests that this proinflammatory response communicates to the brain to facilitate the reduction. This idea is further supported by the fact that treatment with either probiotic or synbiotic attenuated the proinflammatory intestinal changes in the males, correlating with no reduction in plaque load compared to untreated controls. Dysbiosis due to antibiotics treatment resulted in a significant increase in both CD4^+^CD25^+^ T cells and FoxP3 expressing CD4^+^CD25^+^ T regulatory cells in male wild type and *App^NL-G-F^* mice. It is intriguing what role FoxP3 expression in these cells might play, as it is generally believed that CD4+CD25+ T cells expressing the transcription factor, FoxP3 (Tregs), are involved in regulatory functions and suppression of proinflammatory cytokines. Therefore, it is possible that these cells are activation-induced FoxP3^+^ T effector cells lacking regulatory or suppressive functions and may partially contribute to the proinflammatory IL-2, IL-5, IFN-γ, IL-17A, and IL-17F colonic cytokine profile observed in male wild type and *App^NL-G-F^* mice. These assessments indicate that male versus female gut microbiome manipulations have sex-selective effects on peripheral immune cell phenotype that correspond to differing changes in the brain.

In conclusion, these results demonstrate that transiently depleting gut bacteria with antibiotic treatment has a long-term effect on the brain. More importantly, it appears that probiotic intervention alone, and not following an antibiotic pretreatment, is particularly useful in females for improving multiple AD parameters. Therefore, considerations for therapeutic gut microbiome manipulation in AD may need to consider the basal gut microbiota composition and sex. 

## Figures and Tables

**Table 1:** Reagents used for flow cytometry.

Dye/Antibody	Clone	Fluorochrome	Source
Fixable/Viability dye		BV510	Tonbobio
Fc Block			Biolegend
CD45	30F11	PErCPCY5-5	Biolegend
CD3	17A2	FITC	Biolegend
CD4	RM4-5	BV605	Biolegend
CD25	PC61	PE	Biolegend
CD28	37.51	BV421	Biolegend
CD62L	MEL-14	PECY7	Biolegend
CD69	H1.2F3	APC	Biolegend
CD11b	M1/70	PE	Biolegend
CD14		PECY7	Biolegend
CD36		APC	Biolegend
F4/80	BM8	BV421	Biolegend
LY6C	HK1-4	APC/FIRE750	Biolegend
CD4	RM4-5	BV605	Biolegend
CD25	PC61	APC	Biolegend
FOXP3	FJK-16s	PE	eBioscience

**Table 2:** Primers sequences associated with the seven PCR amplicons covering nine variable regions in the bacterial 16S rRNA gene.

Name	Sequence
V1-V2_F	TCGTCGGCAGCGTCAGAGTTTGATCCTGGCTCAG
V2_F	TCGTCGGCAGCGTCAGYGGCGIACGGGTGAGTAA
V3_2_F	TCGTCGGCAGCGTCCCTACGGGAGGCAGCAG
V4_F	TCGTCGGCAGCGTCGTGCCAGCMGCCGCGGTAA
V5-V6_F	TCGTCGGCAGCGTCAGGATTAGATACCCTGGTA
V6_1_F	TCGTCGGCAGCGTCAAACTCAAAKGAATTGACGG
V7-V8_F	TCGTCGGCAGCGTCGYAACGAGCGCAACCC
V1-V2_R	GTCTCGTGGGCTCGGTGCTGCCTCCCGTAGGAGT
V2_R	GTCTCGTGGGCTCGGCYIACTGCTGCCTCCCGTAG
V3_2_R	GTCTCGTGGGCTCGGGTATTACCGCGGCTGCTGG
V4_R	GTCTCGTGGGCTCGGGGACTACHVGGGTWTCTAAT
V5-V6_R	GTCTCGTGGGCTCGGCRRCACGAGCTGACGAC
V6_1_R	GTCTCGTGGGCTCGGACGAGCTGACGACARCCATG
V7-V8_R	GTCTCGTGGGCTCGGGACGGGCGGTGWGTRC

## Data Availability

All data generated or analyzed during this study are included in this published article and its supplementary information files.

## Supplementary Materials

The following are available online at https://www.mdpi.com/article/10.3390/cells10092370/s1, Figure S1: Microbiome difference in the fecal samples of C57BL/6J (WT) and *App^NL-G-F^* female and male mice, Figure S2: Effect of probiotic and antibiotics treatments on intestinal permeability in female and male C57BL/6J (WT) and *App^NL-G-F^* mice, Figure S3: Quantification of Th1, Th2, and Th17 cytokines in the serum of female C57BL/6J (WT) and *App^NL-G-F^* mice, Figure S4: Quantification of Th1, Th2, and Th17 cytokines in the serum of male C57BL/6J (WT) and *App^NL-G-F^* mice.

## Abbreviations

AD	Alzheimer’s disease
CNS	Central nervous system
ENS	Enteric nervous system
Veh	Vehicle
WT	Wild type
ABX	Antibiotics
Syn	Synbiotic (Antibiotics plus probiotics)
CFU	colony forming units
Iba1	Ionized calcium binding adaptor molecule 1
GFAP	Glial fibrillary acidic protein
Aβ	Amyloid beta
PBS	Phosphate buffered saline
ELISA	Enzyme-linked immunosorbent assay
BCA	Bicinchoninic acid
SEM	standard error of the mean
ANOVA	analysis of variance
VSL#3	commercially available probiotic containing eight type of bacteria
APP	amyloid precursor protein
LPS	Lipopolysaccharide

## References

[1] Clayton T.A., Baker D., Lindon J.C., Everett J.R., Nicholson J.K. (2009). Pharmacometabonomic identification of a significant host-microbiome metabolic interaction affecting human drug metabolism. Proc. Natl. Acad. Sci. USA.

[2] Saha J.R., Butler V.P., Neu H.C., Lindenbaum J. (1983). Digoxin-inactivating bacteria: Identification in human gut flora. Science.

[3] Dalile B., Van Oudenhove L., Vervliet B., Verbeke K. (2019). The role of short-chain fatty acids in microbiota-gut-brain communication. Nat. Rev. Gastroenterol. Hepatol..

[4] Silva Y.P., Bernardi A., Frozza R.L. (2020). The Role of Short-Chain Fatty Acids From Gut Microbiota in Gut-Brain Communication. Front. Endocrinol..

[5] Antonini M., Lo Conte M., Sorini C., Falcone M. (2019). How the Interplay Between the Commensal Microbiota, Gut Barrier Integrity, and Mucosal Immunity Regulates Brain Autoimmunity. Front. Endocrinol..

[6] Ahern P.P., Maloy K.J. (2020). Understanding immune-microbiota interactions in the intestine. Immunology.

[7] Cheng H.Y., Ning M.X., Chen D.K., Ma W.T. (2019). Interactions Between the Gut Microbiota and the Host Innate Immune Response Against Pathogens. Front. Endocrinol..

[8] Jandhyala S.M., Talukdar R., Subramanyam C., Vuyyuru H., Sasikala M., Nageshwar Reddy D. (2015). Role of the normal gut microbiota. World J. Gastroenterol..

[9] Brennan C.A., Garrett W.S. (2016). Gut Microbiota, Inflammation, and Colorectal Cancer. Annu. Rev. Microbiol..

[10] Lavelle A., Sokol H. (2020). Gut microbiota-derived metabolites as key actors in inflammatory bowel disease. Nat. Rev. Gastroenterol. Hepatol..

[11] Zhu S., Jiang Y., Xu K., Cui M., Ye W., Zhao G., Jin L., Chen X. (2020). The progress of gut microbiome research related to brain disorders. J. Neuroinflamm..

[12] Carding S., Verbeke K., Vipond D.T., Corfe B.M., Owen L.J. (2015). Dysbiosis of the gut microbiota in disease. Microb. Ecol. Health Dis..

[13] Mulle J.G., Sharp W.G., Cubells J.F. (2013). The gut microbiome: A new frontier in autism research. Curr. Psychiatry Rep..

[14] Kowalski K., Mulak A. (2019). Brain-Gut-Microbiota Axis in Alzheimer’s Disease. J. Neurogastroenterol. Motil..

[15] Sochocka M., Donskow-Lysoniewska K., Diniz B.S., Kurpas D., Brzozowska E., Leszek J. (2019). The Gut Microbiome Alterations and Inflammation-Driven Pathogenesis of Alzheimer’s Disease-a Critical Review. Mol. Neurobiol..

[16] Vogt N.M., Kerby R.L., Dill-McFarland K.A., Harding S.J., Merluzzi A.P., Johnson S.C., Carlsson C.M., Asthana S., Zetterberg H., Blennow K. (2017). Gut microbiome alterations in Alzheimer’s disease. Sci. Rep..

[17] Heneka M.T., Carson M.J., El Khoury J., Landreth G.E., Brosseron F., Feinstein D.L., Jacobs A.H., Wyss-Coray T., Vitorica J., Ransohoff R.M. (2015). Neuroinflammation in Alzheimer’s disease. Lancet Neurol..

[18] Cerovic M., Forloni G., Balducci C. (2019). Neuroinflammation and the Gut Microbiota: Possible Alternative Therapeutic Targets to Counteract Alzheimer’s Disease?. Front. Aging Neurosci..

[19] McCoy K.D., Burkhard R., Geuking M.B. (2019). The microbiome and immune memory formation. Immunol. Cell Biol..

[20] Wu H.J., Ivanov I.I., Darce J., Hattori K., Shima T., Umesaki Y., Littman D.R., Benoist C., Mathis D. (2010). Gut-residing segmented filamentous bacteria drive autoimmune arthritis via T helper 17 cells. Immunity.

[21] Lee Y.K., Menezes J.S., Umesaki Y., Mazmanian S.K. (2011). Proinflammatory T-cell responses to gut microbiota promote experimental autoimmune encephalomyelitis. Proc. Natl. Acad. Sci. USA.

[22] Atarashi K., Tanoue T., Shima T., Imaoka A., Kuwahara T., Momose Y., Cheng G., Yamasaki S., Saito T., Ohba Y. (2011). Induction of colonic regulatory T cells by indigenous Clostridium species. Science.

[23] Fung T.C., Olson C.A., Hsiao E.Y. (2017). Interactions between the microbiota, immune and nervous systems in health and disease. Nat. Neurosci..

[24] Ericsson A.C., Franklin C.L. (2015). Manipulating the Gut Microbiota: Methods and Challenges. ILAR J..

[25] Kaur H., Golovko S., Golovko M.Y., Singh S., Darland D.C., Combs C.K. (2020). Effects of Probiotic Supplementation on Short Chain Fatty Acids in the AppNL-G-F Mouse Model of Alzheimer’s Disease. J. Alzheimer’s Dis. JAD.

[26] Kaur H., Nagamoto-Combs K., Golovko S., Golovko M.Y., Klug M.G., Combs C.K. (2020). Probiotics ameliorate intestinal pathophysiology in a mouse model of Alzheimer’s disease. Neurobiol. Aging.

[27] Vanderpool C., Yan F., Polk D.B. (2008). Mechanisms of probiotic action: Implications for therapeutic applications in inflammatory bowel diseases. Inflamm. Bowel Dis..

[28] Yan F., Polk D.B. (2011). Probiotics and immune health. Curr. Opin. Gastroenterol..

[29] Saito T., Matsuba Y., Mihira N., Takano J., Nilsson P., Itohara S., Iwata N., Saido T.C. (2014). Single App knock-in mouse models of Alzheimer’s disease. Nat. Neurosci..

[30] Chan Y.K., El-Nezami H., Chen Y., Kinnunen K., Kirjavainen P.V. (2016). Probiotic mixture VSL#3 reduce high fat diet induced vascular inflammation and atherosclerosis in ApoE(-/-) mice. AMB Express.

[31] Reagan-Shaw S., Nihal M., Ahmad N. (2008). Dose translation from animal to human studies revisited. FASEB J. Off. Publ. Fed. Am. Soc. Exp. Biol..

[32] Zmora N., Zilberman-Schapira G., Suez J., Mor U., Dori-Bachash M., Bashiardes S., Kotler E., Zur M., Regev-Lehavi D., Brik R.B. (2018). Personalized Gut Mucosal Colonization Resistance to Empiric Probiotics Is Associated with Unique Host and Microbiome Features. Cell.

[33] Schriefer A.E., Cliften P.F., Hibberd M.C., Sawyer C., Brown-Kennerly V., Burcea L., Klotz E., Crosby S.D., Gordon J.I., Head R.D. (2018). A multi-amplicon 16S rRNA sequencing and analysis method for improved taxonomic profiling of bacterial communities. J. Microbiol. Methods.

[34] Nagamoto-Combs K., Manocha G.D., Puig K., Combs C.K. (2016). An improved approach to align and embed multiple brain samples in a gelatin-based matrix for simultaneous histological processing. J. Neurosci. Methods.

[35] Manocha G.D., Floden A.M., Miller N.M., Smith A.J., Nagamoto-Combs K., Saito T., Saido T.C., Combs C.K. (2019). Temporal progression of Alzheimer’s disease in brains and intestines of transgenic mice. Neurobiol. Aging.

[36] Thevaranjan N., Puchta A., Schulz C., Naidoo A., Szamosi J.C., Verschoor C.P., Loukov D., Schenck L.P., Jury J., Foley K.P. (2018). Age-Associated Microbial Dysbiosis Promotes Intestinal Permeability, Systemic Inflammation, and Macrophage Dysfunction. Cell Host Microbe.

[37] Tilg H., Zmora N., Adolph T.E., Elinav E. (2020). The intestinal microbiota fuelling metabolic inflammation. Nat. Rev. Immunol..

[38] Pervolaraki E., Hall S.P., Foresteire D., Saito T., Saido T.C., Whittington M.A., Lever C., Dachtler J. (2019). Insoluble Abeta overexpression in an App knock-in mouse model alters microstructure and gamma oscillations in the prefrontal cortex, affecting anxiety-related behaviours. Dis. Models Mech..

[39] Nelson P.T., Alafuzoff I., Bigio E.H., Bouras C., Braak H., Cairns N.J., Castellani R.J., Crain B.J., Davies P., Del Tredici K. (2012). Correlation of Alzheimer disease neuropathologic changes with cognitive status: A review of the literature. J. Neuropathol. Exp. Neurol..

[40] Dodiya H.B., Kuntz T., Shaik S.M., Baufeld C., Leibowitz J., Zhang X., Gottel N., Zhang X., Butovsky O., Gilbert J.A. (2019). Sex-specific effects of microbiome perturbations on cerebral Abeta amyloidosis and microglia phenotypes. J. Exp. Med..

[41] Gao H., Shu Q., Chen J., Fan K., Xu P., Zhou Q., Li C., Zheng H. (2019). Antibiotic Exposure Has Sex-Dependent Effects on the Gut Microbiota and Metabolism of Short-Chain Fatty Acids and Amino Acids in Mice. mSystems.

[42] Serrano-Pozo A., Muzikansky A., Gomez-Isla T., Growdon J.H., Betensky R.A., Frosch M.P., Hyman B.T. (2013). Differential relationships of reactive astrocytes and microglia to fibrillar amyloid deposits in Alzheimer disease. J. Neuropathol. Exp. Neurol..

[43] Sanmarco L.M., Wheeler M.A., Gutierrez-Vazquez C., Polonio C.M., Linnerbauer M., Pinho-Ribeiro F.A., Li Z., Giovannoni F., Batterman K.V., Scalisi G. (2021). Gut-licensed IFNgamma(+) NK cells drive LAMP1(+)TRAIL(+) anti-inflammatory astrocytes. Nature.

[44] Jang S.E., Lim S.M., Jeong J.J., Jang H.M., Lee H.J., Han M.J., Kim D.H. (2018). Gastrointestinal inflammation by gut microbiota disturbance induces memory impairment in mice. Mucosal Immunol..

[45] Grazul H., Kanda L.L., Gondek D. (2016). Impact of probiotic supplements on microbiome diversity following antibiotic treatment of mice. Gut Microbes.

[46] Atarashi K., Tanoue T., Oshima K., Suda W., Nagano Y., Nishikawa H., Fukuda S., Saito T., Narushima S., Hase K. (2013). Treg induction by a rationally selected mixture of Clostridia strains from the human microbiota. Nature.

[47] Ivanov I.I., Atarashi K., Manel N., Brodie E.L., Shima T., Karaoz U., Wei D., Goldfarb K.C., Santee C.A., Lynch S.V. (2009). Induction of intestinal Th17 cells by segmented filamentous bacteria. Cell.

[48] Bolotin A., de Wouters T., Schnupf P., Bouchier C., Loux V., Rhimi M., Jamet A., Dervyn R., Boudebbouze S., Blottiere H.M. (2014). Genome Sequence of “Candidatus Arthromitus” sp. Strain SFB-Mouse-NL, a Commensal Bacterium with a Key Role in Postnatal Maturation of Gut Immune Functions. Genome Announc..

[49] Stadlbauer V., Engertsberger L., Komarova I., Feldbacher N., Leber B., Pichler G., Fink N., Scarpatetti M., Schippinger W., Schmidt R. (2020). Dysbiosis, gut barrier dysfunction and inflammation in dementia: A pilot study. BMC Geriatr..

[50] Chen C., Ahn E.H., Kang S.S., Liu X., Alam A., Ye K. (2020). Gut dysbiosis contributes to amyloid pathology, associated with C/EBPbeta/AEP signaling activation in Alzheimer’s disease mouse model. Sci. Adv..

[51] Wexler H.M. (2007). Bacteroides: The good, the bad, and the nitty-gritty. Clin. Microbiol. Rev..

[52] Henke M.T., Kenny D.J., Cassilly C.D., Vlamakis H., Xavier R.J., Clardy J. (2019). Ruminococcus gnavus, a member of the human gut microbiome associated with Crohn’s disease, produces an inflammatory polysaccharide. Proc. Natl. Acad. Sci. USA.

[53] Pfeiffer N., Desmarchelier C., Blaut M., Daniel H., Haller D., Clavel T. (2012). Acetatifactor muris gen. nov., sp. nov., a novel bacterium isolated from the intestine of an obese mouse. Arch. Microbiol..

[54] Parker B.J., Wearsch P.A., Veloo A.C.M., Rodriguez-Palacios A. (2020). The Genus Alistipes: Gut Bacteria With Emerging Implications to Inflammation, Cancer, and Mental Health. Front. Immunol..

[55] Kundu P., Torres E.R.S., Stagaman K., Kasschau K., Okhovat M., Holden S., Ward S., Nevonen K.A., Davis B.A., Saito T. (2021). Integrated analysis of behavioral, epigenetic, and gut microbiome analyses in App(NL-G-F), App(NL-F), and wild type mice. Sci. Rep..

[56] Re F., Strominger J.L. (2003). Separate functional domains of human MD-2 mediate Toll-like receptor 4-binding and lipopolysaccharide responsiveness. J. Immunol..

[57] Belkaid Y., Hand T.W. (2014). Role of the microbiota in immunity and inflammation. Cell.

[58] Cheng H., Guan X., Chen D., Ma W. (2019). The Th17/Treg Cell Balance: A Gut Microbiota-Modulated Story. Microorganisms.

