# Supplementary material for: Sex-Dependent Effects of Intestinal Microbiome Manipulation in a Mouse Model of Alzheimer’s Disease
Source: Cells. 2021 Sep 9;10(9):2370. doi: 10.3390/cells10092370 (PMC8469717; doi:10.3390/cells10092370)
Supplement: Supplementary file 1 [file cells-10-02370-s001.zip › cells-1363318-supplementary.pdf]

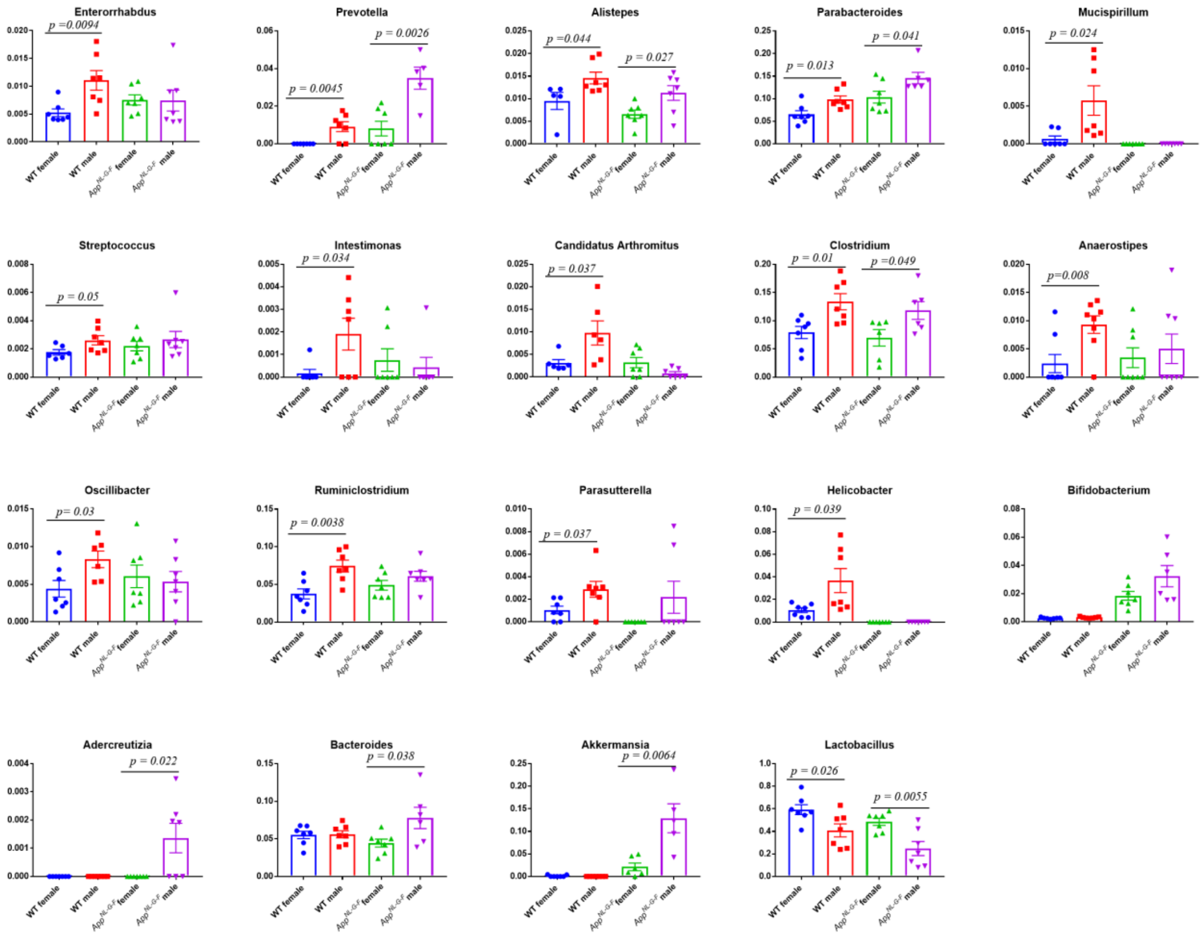

Figure S1. Microbiome difference in the fecal samples of C57BL/6J (WT) and $App^{NL\text{-}G\text{-}F}$ female and male mice. The relative abundance of significantly different bacteria genera in male and female C57BL/6J (WT) and $App^{NL\text{-}G\text{-}F}$ mice are plotted. The differences in the bacterial composition of male vs. female C57BL/6J (WT) and male vs. female $App^{NL\text{-}G\text{-}F}$ mice were determined by unpaired t-test, * $p < 0.05$. Data are presented as mean ± SEM, $n = 7$.

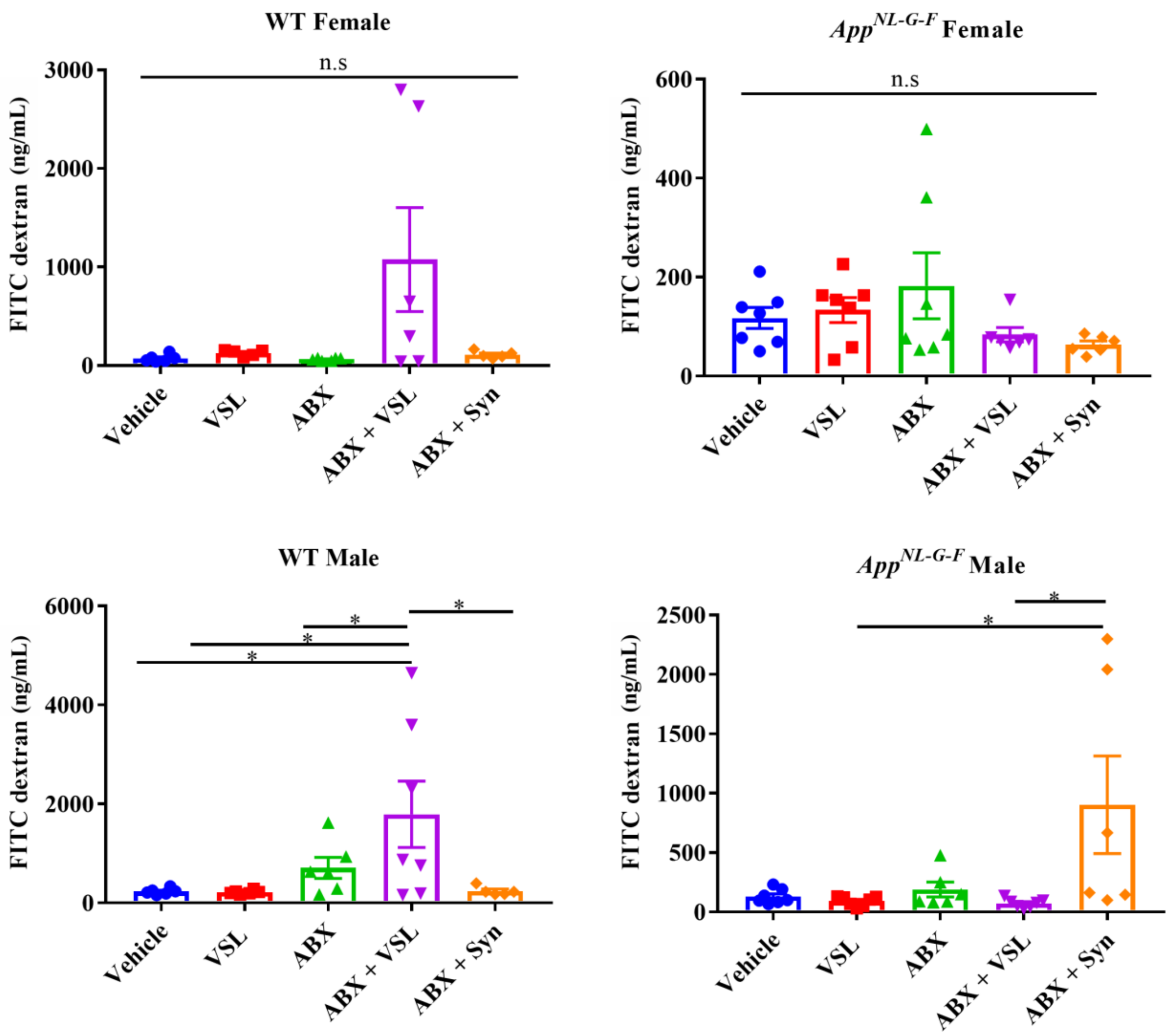

Figure S2. Effect of probiotic and antibiotics treatments on intestinal permeability in female and male C57BL/6J (WT) and *App*$^{NL\text{-}G\text{-}F}$ mice. Permeability assays were performed on female and male C57BL/6J (WT) and *App*$^{NL\text{-}G\text{-}F}$ mice following completion of the treatment paradigm. The concentration of 4 kDa FITC-conjugated dextran was measured in serum 5 h after oral gavage. Data are presented as mean ± SEM. Significant differences were determined by one-way analysis of variance, * $p < 0.05$ ($n = 7$).

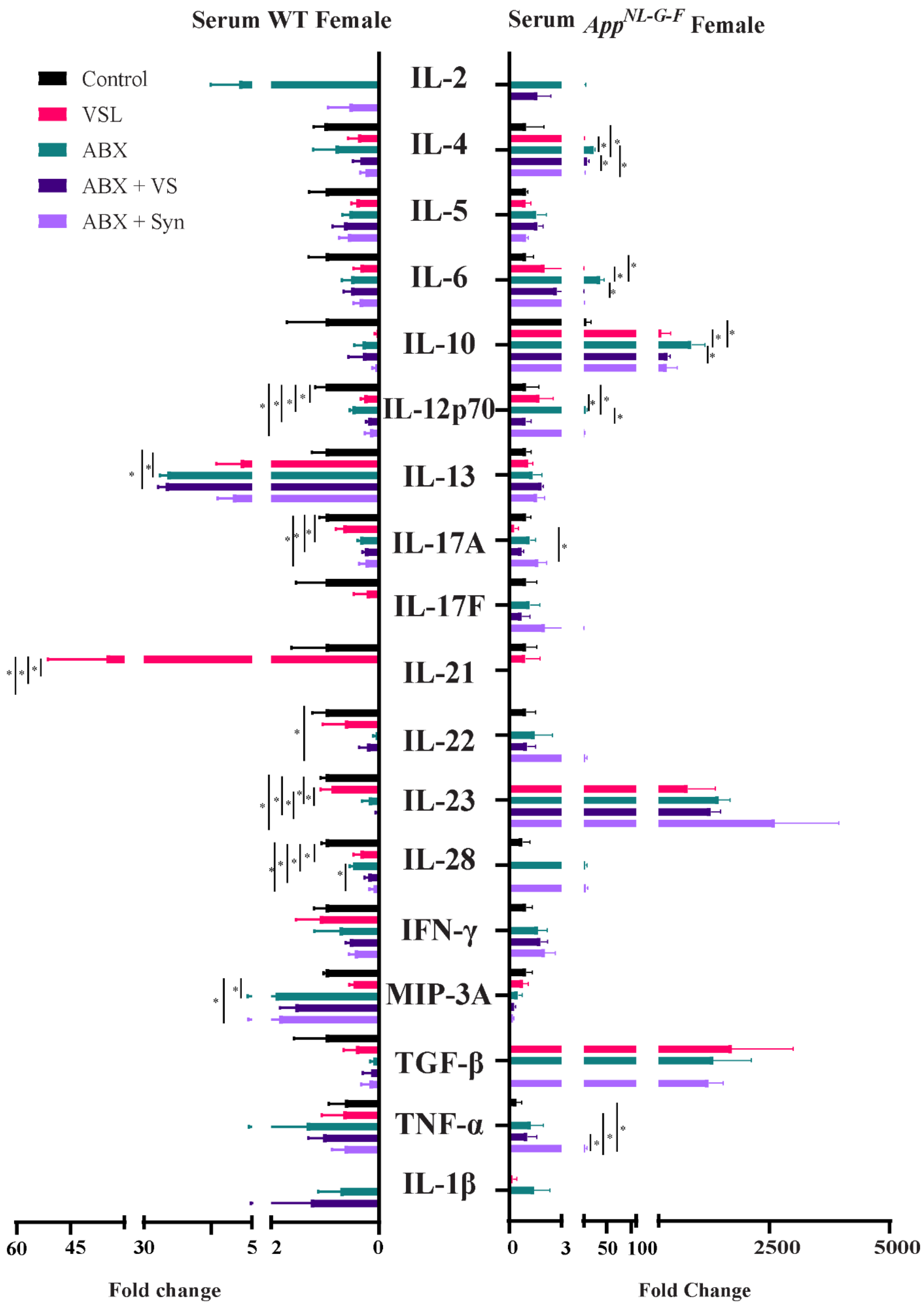

Figure S3. Quantification of Th1, Th2, and Th17 cytokines in the serum of female C57BL/6J (WT) and App^{NL-G-F} mice. Serum cytokines from vehicle, VSL#3, antibiotics (ABX), antibiotics + VSL#3 (ABX + VSL), and antibiotics + VSL#3 + prebiotic (ABX + Syn) female WT mice were measured via commercial slide array. Data are presented as fold change with respect to controls. Significant differences were determined by one-way analysis of variance, * $p < 0.05$.

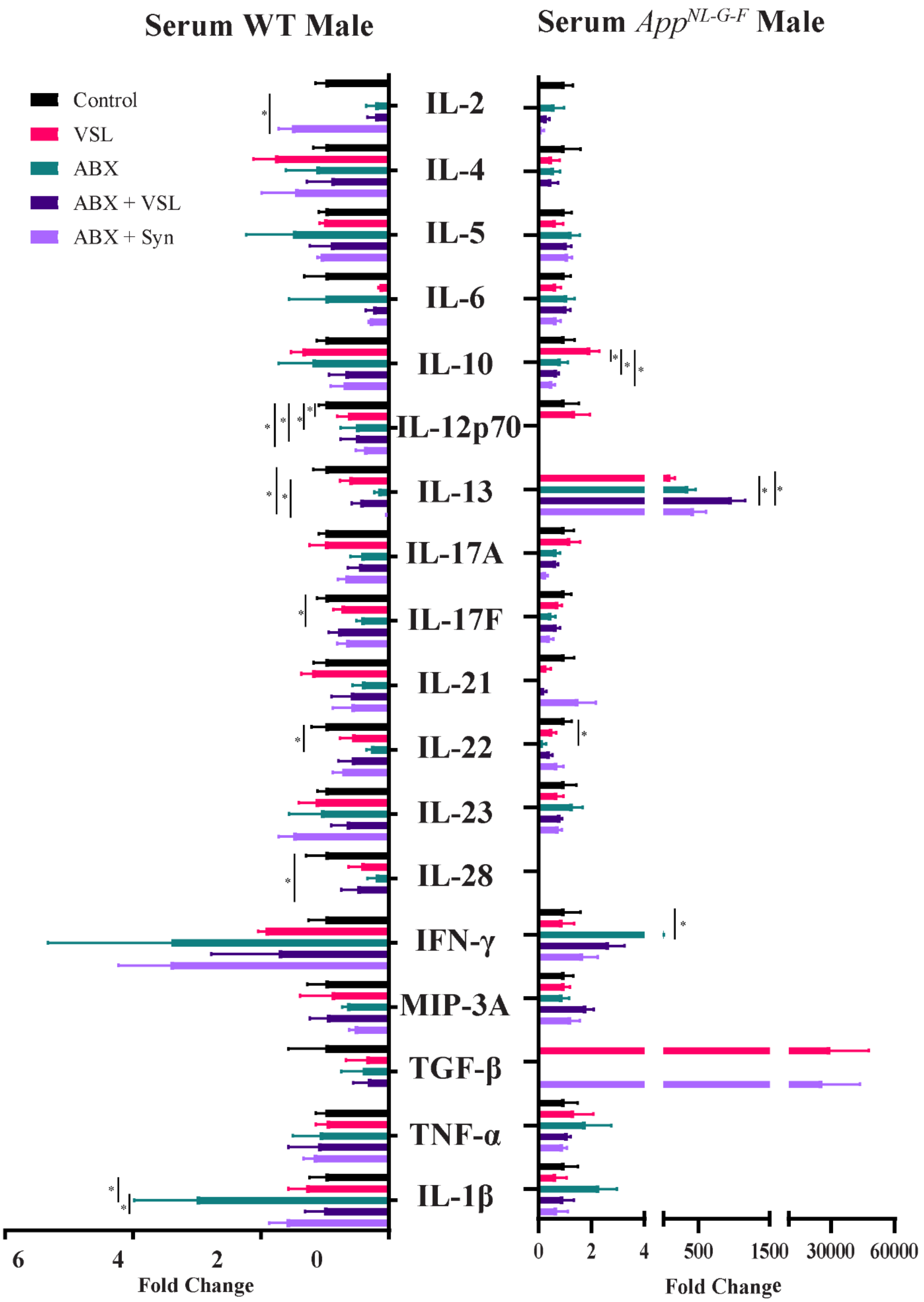

Figure S4. Quantification of Th1, Th2, and Th17 cytokines in the serum of male C57BL/6J (WT) and $App^{NL\text{-}G\text{-}F}$ mice. Serum cytokines from vehicle, VSL#3, antibiotics (ABX), antibiotics + VSL#3 (ABX + VSL), and antibiotics + VSL#3 + prebiotic (ABX + Syn) male WT mice were measured via commercial slide array. Data are presented as fold change with respect to controls ($n = 5$). Significant differences were determined by one-way analysis of variance, * $p < 0.05$.
